# A Multi-Strategy Parrot Optimization Algorithm and Its Application

**DOI:** 10.3390/biomimetics10030153

**Published:** 2025-03-02

**Authors:** Yang Yang, Maosheng Fu, Xiancun Zhou, Chaochuan Jia, Peng Wei

**Affiliations:** College of Electronic and Information Engineering, West Anhui University, Lu’an 237012, China; 03000031@wxc.edu.cn (Y.Y.); zhouxc@wxc.edu.cn (X.Z.); 03000075@wxc.edu.cn (C.J.); 15375373707@163.com (P.W.)

**Keywords:** parrot optimization algorithm, chaotic logistic map, gaussian mutation, barycenter opposition-based learning, industrial refrigeration systems, Himmel Blau’s function, indoor visible light positioning

## Abstract

Intelligent optimization algorithms are crucial for solving complex engineering problems. The Parrot Optimization (PO) algorithm shows potential but has issues like local-optimum trapping and slow convergence. This study presents the Chaotic–Gaussian–Barycenter Parrot Optimization (CGBPO), a modified PO algorithm. CGBPO addresses these problems in three ways: using chaotic logistic mapping for random initialization to boost population diversity, applying Gaussian mutation to updated individual positions to avoid premature local-optimum convergence, and integrating a barycenter opposition-based learning strategy during iterations to expand the search space. Evaluated on the CEC2017 and CEC2022 benchmark suites against seven other algorithms, CGBPO outperforms them in convergence speed, solution accuracy, and stability. When applied to two practical engineering problems, CGBPO demonstrates superior adaptability and robustness. In an indoor visible light positioning simulation, CGBPO’s estimated positions are closer to the actual ones compared to PO, with the best coverage and smallest average error.

## 1. Introduction

With the increasing complexity of real-world problems, traditional methods have encountered numerous limitations. Against such challenging circumstances, intelligent optimization algorithms have progressively emerged. They stem from the observation and emulation of phenomena in nature and the intelligent behaviors of organisms. Initially, such algorithms were rudimentary and merely simulated biological evolution; examples include the Simulated Annealing (SA) algorithm [1], the Genetic Algorithm (GA) [2], the Particle Swarm Optimization (PSO) algorithm [3], and the Ant Colony Optimization (ACO) algorithm [4].

With the rapid development of science and technology, intelligent optimization algorithms have evolved and diversified into numerous types. These include the Bacterial Foraging Optimization (BFO) [5], Artificial Bee Colony (ABC) [6], Cuckoo Search (CS) [7], Bat Algorithm (BA) [8], Moth Flame Optimization (MFO) [9], Pigeon Swarm Optimization Algorithm (PSOA) [10], Spider Monkey Optimization (SMO) [11], Seagull Optimization algorithm (SOA) [12], Remora Optimization Algorithm (ROA) [13], Black Smoke Swallow Optimization algorithm (STOA) [14], and Gray Wolf Optimization (GWO) [15] algorithms. In recent years, new ones like the Harris Hawks Optimization (HHO) [16], Catch fish optimization algorithm (CFOA) [17], Pelican Optimization Algorithm (POA) [18], and Crayfish Optimization Algorithm (COA) [19] have also emerged.

Most intelligent optimization algorithms simulate the behaviors and habits of natural organisms to efficiently solve complex problems. For instance, authors in [20] proposed a Hybrid Whale Particle Swarm Optimization (HWPSO) algorithm, leveraging the “forced” whale and “capping” phenomenon. Evaluated in tasks like determining operational amplifier circuit size, minimizing micro-electro-mechanical system switch pull-in voltage, and reducing differential amplifier random offset, the HWPSO operated efficiently and improved optimal values significantly. In [21], an Improved Whale Optimization Algorithm (IWOA) was proposed, combining crossover and mutation from the Differential Evolution (DE) algorithm and introducing a cloud-adaptive inertia weight. This algorithm was applied to truss structure optimization for 52-bar planar and 25-bar spatial trusses. Authors of Ref. [22] enhanced the Seagull Optimization Algorithm with Levy flight (LSOA), achieving good results in path-planning problems. Moreover, Ref. [23] introduced a Multi-strategy Golden Jackal Optimization (MGJO) algorithm. It initialized the population via chaotic mapping, adopted a non-linear dynamic inertia weight and used Cauchy mutation to boost population diversity, effectively estimating parameters of the non-linear Muskingum model.

The Parrot Optimization (PO) [24], proposed by J. Lian et al. in 2024, is inspired by parrots’ learning behaviors. It emulates parrots’ foraging, lingering, communication, and wariness of strangers to extensively search for the optimal solution in the search space. Yet, when handling complex problems with high-dimensional, multi-modal, and non-linear objective functions, the PO algorithm’s search efficiency and solution accuracy may suffer. It often converges prematurely to local optima or fails to fully explore the search space, hindering the discovery of the global optimum. Furthermore, lacking an adaptive parameter adjustment mechanism, the PO algorithm’s parameters cannot be automatically tuned. Manual parameter adjustment for various optimization problems is thus required, decreasing the algorithm’s convergence rate.

To address PO’s drawbacks, like becoming trapped in local optima and slow convergence, we developed a multi-strategy PO algorithm named CGBPO. Firstly, chaotic logistic mapping replaces traditional random initialization. This distributes the initial population more evenly and widely, preventing individuals from clustering in specific search space areas [25]. It boosts population diversity and enhances global search ability. Secondly, Gaussian mutation is applied to updated individual positions [26]. Everyone has a chance to generate new, relatively random positions nearby, breaking local convergence and increasing diversity. Thirdly, after each iteration, we calculate the centroid of the current population and generate the corresponding opposite solution [27]. Then, we explore the search space from the direction opposite to the current individuals based on the centroid, guiding the population to more promising areas. This makes the search more directional, comprehensive, and better at handling complex high-dimensional and multimodal optimization problems.

To validate the CGBPO algorithm, 30 independent experiments were carried out in CEC2017 [28], CEC2022 [29], and two engineering problems, followed by a comparison with seven swarm intelligence algorithms. The results show that the three strategies in CGBPO effectively enhance its performance, notably addressing premature local optimization and slow convergence issues.

The paper is organized as follows: Section 2 overviews the PO algorithm. Section 3 elaborates on the proposed CGBPO with multiple strategies. Section 4 and Section 5 detail the experimental tests of CGBPO on CEC2017 and CEC2022 benchmarks and analyze the results. Section 6 presents the comparative tests of CGBPO for engineering problems. Section 7 describes the application of CGBPO in the indoor visible-light-positioning system. Finally, Section 8 summarizes the study and outlines future research directions.

## 2. Parrot Optimization Algorithm

The PO algorithm primarily encompasses the four behaviors detailed below.

### 2.1. Foraging Behavior

Through observing the location of food or their owner’s position, parrots estimate the approximate location of food and then fly toward it. Thus, the movement of parrots is modeled using the following formula:

(1) $$X_{i}^{t+1}=(X_{i}^{t}-X_{best})\cdot Levy(dim)+rand(0,1)\cdot {\left(1-\frac{t}{Max_{iter}}\right)}^{\frac{2t}{Max_{iter}}}))\cdot X_{mean}^{t}$$

(2) $$X_{mean}^{t}=\frac{1}{N}\;\sum_{k=1}^{N}X_{k}^{t}$$

(3) $$\left\{\begin{array}{c} Levy(dim)=\frac{\mu \cdot \sigma }{{\left|\nu \right|}^{\frac{1}{\gamma }}}\\ \mu ∼N(0,dim)\\ \nu ∼N(0,dim)\\ \sigma ={\left(\frac{\Gamma \left(1+\gamma \right)\cdot sin\left(\frac{\pi \gamma }{2}\right)}{\Gamma \left(\frac{1+\gamma }{2}\right)\cdot \gamma 2^{\frac{1+\gamma }{2}}}\right)}^{\gamma +1} \end{array}\right.$$

where $X_{i}^{t}$ stands for the current position; $X_{i}^{t+1}$ symbolizes the updated position; $Max_{iter}$ is the maximum number of iterations; $X_{mean}^{t}$ represents the average position of the current population, as defined in Equation (2); Levy() represents the Levy distribution, as defined in Equation (3), which serves to depict the flight of the parrots; γ is given a value of 1.5, which is used to describe the flight of the parrots; $X_{best}$ represents the current optimal position; t represents the current iteration; $\left(X_{i}^{t}-X_{best})\cdot Levy(dim\right)$ represents the movement based on the relative position to the owner, and $rand(0,1)\cdot {\left(1-\frac{t}{Max_{iter}}\right)}^{\frac{2t}{Max_{iter}}}))\cdot X_{mean}^{t}$ represents determination of the location of food more precisely through observing the positions of the whole population.

### 2.2. Staying Behavior

Modeling the behavior of parrots staying randomly on different parts of their owner’s body allows for the incorporation of randomness into the search process:

(4) $$X_{i}^{\left(t+1\right)}=X_{i}^{t}+X_{best}\cdot Levy(dim)+rand(0,1)\cdot ones(1,dim)$$

where $X_{best}\cdot Levy(dim)$ indicates the process of flying toward the owner, and rand(0,1)⋅ones(1,dim) symbolizes randomly stopping on a certain part of the owner’s body.

### 2.3. Communicating Behavior

Parrots are inherently gregarious and often communicate within their groups, either by flying towards the flock or communicating while staying away from it. In the PO algorithm, it is assumed that these two behaviors have an equal probability of occurrence, and the average position of the current population is taken as the representation of the flock’s center. This behavior is modeled as follows:

(5) $$X_{i}^{\left(t+1\right)}=\left\{\begin{array}{c} 0.2\cdot rand(0,1)\cdot (1-\frac{t}{Max_{iter}})\cdot (X_{i}^{t}-X_{mean}^{t}),P\leq 0.5\\ 0.2\cdot rand(0,1)\cdot exp(-\frac{t}{rand(0,1)\cdot Max_{iter}}),P>0.5 \end{array}\right.$$

where $0.2\cdot rand(0,1)\cdot (1-\frac{t}{Max_{iter}})\cdot (X_{i}^{t}-X_{mean}^{t})$ represents the course of an individual becoming part of a parrot flock for communication, while $0.2\cdot rand(0,1)\cdot exp(-\frac{t}{rand(0,1)\cdot Max_{iter}})$ represents the situation where an individual takes off right after communicating. Both behaviors are realized through generating a random number within the interval [0, 1].

### 2.4. Fear of Strangers Behavior

Parrots have a natural fear of unfamiliar individuals and, so, will stay away from strangers and seek protection from their owners. This behavior is modeled as follows:

(6) $$\begin{array}{l} X_{i}^{t+1}=X_{i}^{t}+rand(0,1)\cdot cos(0.5\pi \cdot \frac{t}{Max_{iter}})\cdot (X_{best}-X_{i}^{t})\\ -cos(rand(0,1)\cdot \pi )\cdot {\left(\frac{t}{Max_{iter}}\right)}^{\frac{2}{Max_{iter}}}))\cdot (X_{i}^{t}-X_{best}) \end{array}$$

where $rand(0,1)\cdot cos(0.5\pi \cdot \frac{t}{Max_{iter}})\cdot (X_{best}-X_{i}^{t})$ indicates the process of reorienting to fly toward the owner, and $cos(rand(0,1)\cdot \pi )\cdot {\left(\frac{t}{Max_{iter}}\right)}^{\frac{2}{Max_{iter}}}))\cdot (X_{i}^{t}-X_{best})$ symbolizes the procedure of distancing oneself from strangers.

## 3. Multi-Strategy Parrot Optimization Algorithm (CGBPO)

### 3.1. Chaotic Logistic Map Strategy

The chaotic logistic map is defined using a simple recurrence relation, as depicted in Equation (7):

(7) $$x_{i+1}=ax_{i}\left(1-x_{i}\right)$$

In this context, $x_{i}$ stands for the state value in the ith iteration, while $x_{i+1}$ represents the state value in the subsequent iteration. The parameter a serves as a control factor, and its value generally falls within the range of 0 to 4. Additionally, when the value of a is confined to the interval between 0 and 1, as the number of iterations increases, the sequences generated in the subsequent steps will display entirely distinct changing tendencies. Furthermore, when the value of a lies within the approximate interval from 3.57 to 4, the system displays chaotic characteristics. In this case, 4 is selected. For different initial values, the system exhibits chaotic behavior after multiple iterations. More precisely, the sequence of system state values appears to be disorderly and fluctuates in a random manner. Even a minute difference in the initial value will result in completely different trajectories for the subsequent state values as the iterations progress.

There are other common chaotic strategies like the Tent map [30] and the Sine map [31]. The Tent map shows chaotic features in a narrow range around the control parameter value of 2, so its chaotic domain is relatively narrow. The Sine map turns chaotic when the control parameter approaches 1, but the boundaries of its chaotic regime are ambiguous. Compared with them, the logistic map has outstanding randomness and ergodicity. It can evenly cover the defined interval without obvious data aggregation. It is highly sensitive to the initial value; a tiny change can lead to greatly different results after multiple iterations, a key chaotic feature. The Tent and Sine maps are also sensitive to initial conditions, but less so than the logistic map. In the early stages, differences from different initial values are not obvious.

Due to this, the logistic map is used in the optimization algorithm’s initialization and perturbation. It can boost the algorithm’s global search ability, avoid local optima, and enhance overall optimization performance. In the proposed PO method’s initialization, the logistic map sets the initial positions of individual parrots, introducing chaotic perturbations. After iterations, individual search trajectories become more random, breaking away from local optima that the traditional update mode may become stuck in. This improves the algorithm’s global search ability, balances exploration and exploitation, and enhances its optimization ability.

To assess the performance of the selected map strategies, the CEC2022 standard dataset is used with MATLAB(R2023a) simulation software. Three algorithms—CPO, TPO, and SPO—improving the PO algorithm with the chaotic logistic map, Tent map, and Sine map, respectively, are tested. Then, the test results are compared and analyzed. The population size is set at 30, the maximum number of iterations is 300, and each of the three algorithms runs independently 30 times for every test function.

**Figure 1:**
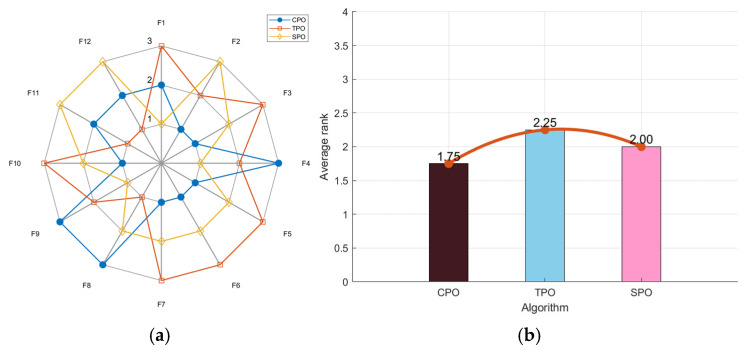
Radar chart (**a**) and ranking chart (**b**) of three algorithms using map strategies.

As shown in Figure 1a, the CPO’s radar chart has the least area fluctuation. CPO ranked first for six functions and second for three functions, indicating highly stable results across multiple runs, unaffected by random factors. In Figure 1b, the ranking chart, sorted by average fitness values, shows that CPO has the lowest average fitness value. In the comprehensive test, the CPO algorithm obtained better solutions and outperformed the other two algorithms in convergence performance.

### 3.2. Gaussian Mutation Strategy

Gaussian mutation randomly modifies the genetic information of individuals with random perturbation values that adhere to a Gaussian distribution (normal distribution), thereby giving rise to new individuals after the mutation process. The Gaussian mutation function is presented in Equation (8):

(8) $$x_{i}=N\left(\mu ,\sigma \right)$$

The Ν function is employed to generate random numbers that comply with the normal distribution. In this context, μ and σ serve as the mean and standard deviation of the Gaussian function, respectively, as illustrated in Equations (9) and (10):

(9) $$\mu =\frac{lb+ub}{2}$$

(10) $$\sigma =\frac{ub-lb}{6}$$

Typically, lb symbolizes the lower bound of the variable, while ub represents the upper bound of the variable. The average of the upper and lower bounds is taken as the central position of the normal distribution. This configuration ensures that the generated random mutation values are theoretically distributed in a relatively uniform manner around this value range. Given that it is grounded in a normal distribution, approximately 99.7% of the mutation values will fall within the range of the mean plus or minus three times the standard deviation, that is, $\left[\mu -3\sigma ,\mu +3\sigma \right]$. Consequently, there is a high likelihood that the mutation values will lie within this interval centered around the mean, with a width equivalent to ub−lb (as $3\sigma =\frac{ub-lb}{2}$, extending by this amount on both sides precisely covers the width of the interval). This implies that the degree of dispersion of the mutation is relatively moderate. It will neither cause the new solutions generated by mutation to be overly scattered and distant from the original value range nor make the mutation overly concentrated, thus losing the ability to explore new solution spaces. Instead, it represents a form of exploration that has a certain breadth yet remains controllable within the given upper- and lower-bound intervals. Consequently, when the range of the upper and lower bounds (i.e., the value of ub−lb) is large, σ will also be large, signifying an increase in the degree of dispersion of the mutation. With the mutation operation, a larger value range can be explored more comprehensively, and it is more likely that individuals can break free from local optimal solutions and search for new areas in the solution space that are removed from the current solution. Conversely, if the range of the upper and lower bounds is small, σ will become smaller, the degree of dispersion of the mutation will decrease, and the new solutions generated by the mutation operation will be closer to the current mean position, focusing more on fine-tuning and optimizing the local area within a relatively small interval.

Cauchy mutation [32] and non-uniform mutation [33] are two common mutation strategies in optimization algorithms. Cauchy mutation modifies individual genes using the Cauchy distribution. It randomly samples a value from this distribution and adds it to the original gene value to obtain the mutated one. The Cauchy distribution’s heavy tail means there is a high chance of obtaining a value far from the central value. So, Cauchy mutation is mainly for global search, but it has low local search accuracy. Also, its large mutation values lead to poor stability and fluctuations result.

The probability of non-uniform mutation declines with iterations, aiding global exploration initially and focusing on local development later. The mutation amplitude varies dynamically, large at first then small. Its direction is random and parameter adjustable. Yet, parameter setting is tough and demands much debugging. Its adaptability to different problems is limited with varying effects, has high computational complexity, and may still become trapped in local optima, hindering global optimization.

In contrast, Gaussian mutation is highly stable. Based on the normal distribution, it gives the mutation process direction and concentration. Most mutation values are within a certain range, ensuring the algorithm’s stable convergence. For local research, Gaussian mutation converges fast and accurately, allowing for precise adjustments to the current solution, making it ideal for scenarios requiring local optimization precision.

**Figure 2:**
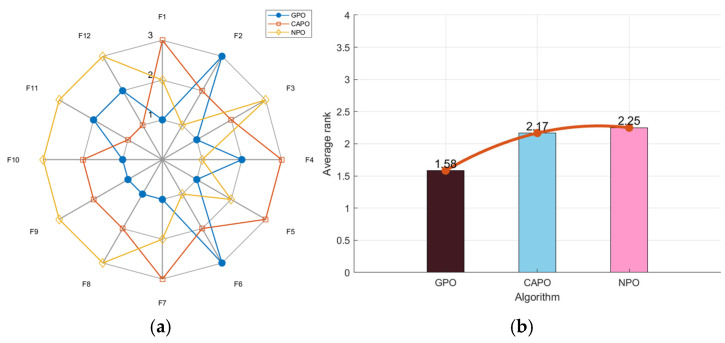
Radar chart (**a**) and ranking chart (**b**) of three algorithms using mutation strategies.

To check the performance of the chosen chaotic strategies, three algorithms—GPO, CAPO, and NPO, which enhance the PO algorithm with Gaussian mutation, Cauchy mutation, and No-uniform mutation, respectively—are used for testing. As shown in Figure 2a, the GPO’s radar chart has the least area fluctuation. GPO ranked first for seven functions and second for three functions, proving its results across multiple runs were highly stable and unaffected by random factors. Notably, GPO had the lowest average fitness value of 1.58 in Figure 2b. In the comprehensive test, GPO performed the best.

### 3.3. Barycenter Opposition-Based Learning Strategy

In this study, barycenter opposition-based learning is adopted. In the initial iterations, as the differences between individuals in the population are relatively large, the generation of mutant parrots allows for more areas to be explored. Meanwhile, in later iterations, although the quantity of individuals in the population decreases, the mutant parrots can still maintain diversity. The barycenter is defined as follows:

Suppose that $\left(m_{1j},m_{2j},\cdots ,m_{nj}\right)$ denotes the values of n parrots in the jth dimension. The population consists of *N* individuals. Then, the barycenter of the parrot population in the jth dimension is given by Equation (11), and the population barycenter is $Z=\left(z_{1},z_{2},\cdots ,z_{j}\right)$.

(11) $$Z_{J}=\frac{x_{1j}+x_{2j}+\cdots +x_{nj}}{n}$$

Barycenter opposition-based mutation: Suppose that $x_{i}=\left(x_{i1},x_{i2},\cdots ,x_{iD}\right)$ is the ith parrot with *D* dimensions. If the selected mutation dimension is the jth dimension, then the barycenter opposition-based solution corresponding to the ith parrot is $x_{op\_i}=\left(x_{op\_i1},x_{op\_i2},\cdots ,x_{op\_iD}\right)$, which is determined using Equation (12):

(12) $$x_{op\_i}=2\times k\times Z_{j}-x_{i}$$

where k is a contraction factor, the value of which is a random figure within the range. During the iteration process, for each parrot in the parrot population, a certain dimension j is selected for mutation. Then, the mutation result is compared with the position of the previous generation, and the better mutation is retained.

Opposition-based learning [34] and elite opposition-based learning [35] are common strategies in optimization algorithms. Opposition-based learning is simple and effective at the start for boosting population diversity, providing more search trajectories and enhancing exploration. But it has a limited way of generating opposite individuals, relying only on individual features and search space boundaries, ignoring other individuals’ distribution and relationships, which may limit its performance in complex scenarios.

Elite opposition-based learning selects elite individuals with high fitness as the core to generate opposite ones, leveraging their high-quality information to find better solutions and speed up convergence. However, focusing on elites reduces population diversity, increasing the risk of premature local optima and missing the global optimum.

In contrast, the Barycenter opposition-based learning strategy uses the population’s overall barycenter information and random contraction adjustment to explore a new solution space opposite the original individuals. It aims to find better solutions different from the current ones, enhancing population diversity, helping the algorithm escape local optima and search for the entire solution space more efficiently.

**Figure 3:**
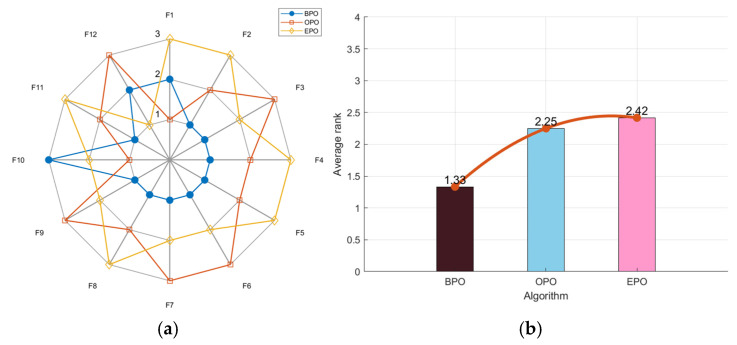
Radar chart (**a**) and ranking chart (**b**) of three algorithms using opposition-based-learning strategies.

To verify the performance of the chosen opposition-based learning strategies, three algorithms—BPO, OPO, and EPO, which enhance the PO algorithm with barycenter opposition-based learning, traditional opposition-based learning, and elite opposition-based learning, respectively—are employed for testing. As shown in Figure 3a, BPO’s radar chart has the least area fluctuation. BPO ranked first for nine functions and second for two functions, demonstrating that its results across multiple runs were highly stable and unaffected by random factors. Notably, BPO had the lowest average fitness value of 1.33 in Figure 3b. In the comprehensive test, BPO performed the best.

### 3.4. Ablation Study of CGBPO

An ablation study was carried out to clarify the contributions of each newly added strategy. The CGBPO algorithm without chaotic mapping (CGBPO1), without Gaussian mutation (CGBPO2), and without barycenter opposition-based learning (CGBPO3), and the original CGBPO algorithm were tested using the CEC2022 standard dataset with MATLAB simulation software. The population size was 30, the maximum iterations were 300, and each algorithm ran independently 30 times for every test function.

**Figure 4:**
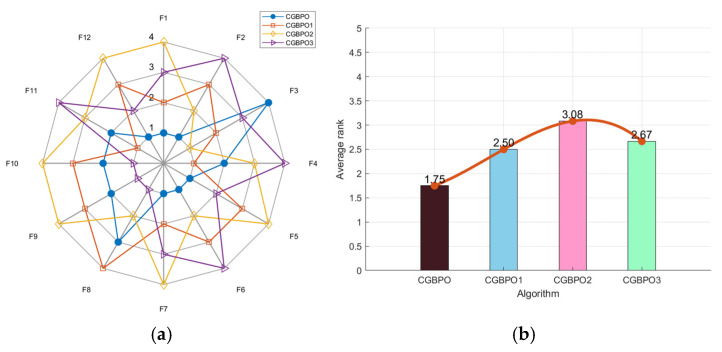
Radar chart (**a**) and ranking chart (**b**) of ablation study.

In Figure 4a, CGBPO’s radar chart had the least area fluctuation, ranking first for six functions and second for four. The radar chart areas of the other three algorithms were much larger, showing the positive effects of the three new strategies on algorithm performance. Notably, in Figure 4b, CGBPO had the lowest average fitness value of 1.33 and the best performance in the comprehensive test. In the ranking chart, CGBPO1 ranked second, CGBPO2 fourth, and CGBPO3 third, indicating that Gaussian mutation contributed most to algorithm improvement.

### 3.5. Pseudo-Code of CGBPO

**Figure 5:**
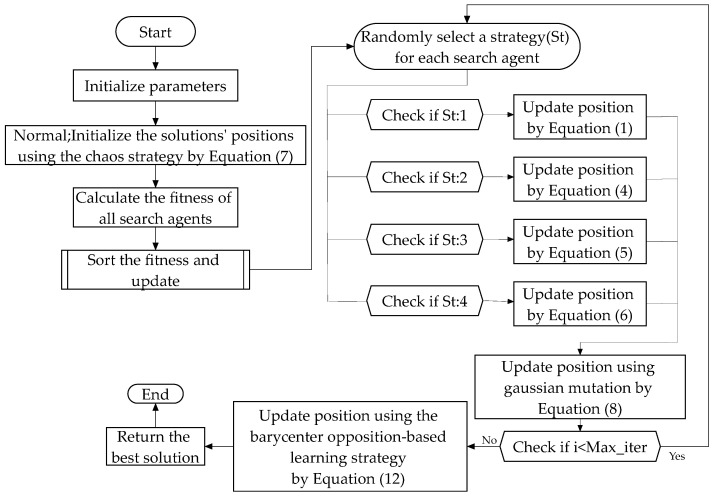
Flowchart of CGBPO.

The comprehensive structure of the CGBPO is presented in Figure 5 and Algorithm 1, which offer a meticulous roadmap for the whole improvement process, encompassing its iterative steps, as well as the utilized search strategies.
**Algorithm 1: Pseudo-Code of CGBPO**1: Initialize the CGBPO parameters2: Initialize the solutions’ positions using the chaos strategy by Equation (7)3: For i = 1: N do4:        Calculate the fitness value of all search agents 5: End6: For i = 1: Max_iter do7:     Find the best position and worst position:8:     For j = 1: N do9:             St = randi([1,4])10:            Behavior 1: Foraging behavior11:            If St == 1 Then12:                 Update position by Equation (1)13:            Behavior 2: Staying behavior14:            Elseif St == 2 Then15:                 Update position by Equation (4)16:            Behavior 3: Communicating behavior17:            Elseif St == 3 Then18:                 Update position by Equation (5)19:            Behavior 4: The fear of strangers’ behavior20:              Elseif St == 4 Then21:                 Update position by Equation (6)22:            End23:              Update position using gaussian mutation by Equation (8)24:     End25:     Generate new solutions using the barycenter opposition-based learning:26: For i = 1: N do27:        Calculate the values of the original function28:        Update position using the barycenter opposition-based learning strategy by Equation (12) 29: End30:      Return the best solution31: End

### 3.6. Comparative Analysis of the Time Complexity Between CGBPO and PO

#### 3.6.1. Time Complexity Analysis of the PO Algorithm

The population initialization operation of PO adopts a simple random initialization method. It generates N individuals with a dimension of dim, and the time complexity is $O\left(N\times \dim\right)$. When calculating the initial fitness values, the objective function values are calculated for N individuals, respectively. Assuming that the time complexity of calculating the objective function once is $O\left(k\right)$ (where k depends on the complexity of the objective function), the time complexity of calculating the initial fitness values is $O\left(N\times k\right)$. The sorting operation uses the sort of function. The time complexity of common sorting algorithms is $O\left(N\log N\right)$. Therefore, the total time complexity of the initialization stage is $O\left(N\times \dim+N\times k+N\log N\right)$.

The PO algorithm has two nested loops in each iteration. The outer loop runs Max_iter times, and the inner loop operates on the N individuals in the population. When updating individuals, calculations such as Levy flight are involved, and its time complexity mainly depends on the dimension dim. Assuming that the time complexity of each individual update operation is $O\left(m\times \dim\right)$ (where m is a constant related to the specific calculation), then the time complexity of individual updates in each iteration is $O\left(N\times m\times \dim\right)$. The boundary control operation also judges and adjusts the dim dimensions of N individuals, and its time complexity is $O\left(N\times \dim\right)$. The time complexities of updating the global optimal solution and sorting the population are $O\left(N\right)$ and $O\left(N\log N\right)$, respectively. Therefore, the time complexity of each iteration is $O\left(N\times m\times \dim+N\times \dim+N+N\log N\right)$, and the time complexity of the entire iteration stage is $O\left(Max\_iter\times \left(N\times m\times \dim+N\times \dim+N+N\log N\right)\right)$.

Combining the initialization and iteration stages, the time complexity of PO is $O\left(N\times \dim+N\times k+N\log N+Max\_iter\times \left(N\times m\times \dim+N\times \dim+N+N\log N\right)\right)$. When N, dim, and Max_iter are large, by ignoring the lower-order terms, the main time complexity can be approximated as $O\left(Max\_iter\times N\times \dim\right)$.

#### 3.6.2. Time Complexity Analysis of the CGBPO Algorithm

The CGBPO algorithm uses chaotic initialization. This function generates chaotic values and maps them to the search space to generate N individuals with a dimension of dim. The time complexity is $O\left(N\times \dim\right)$. The subsequent operations, such as calculating fitness values and sorting are the same as those of PO. Therefore, the total time complexity of the initialization stage is $O\left(N\times \dim+N\times k+N\log N\right)$.

During the iteration process of CGBPO, Gaussian mutation and centroid-based opposition-learning operations are added. Each mutation operation involves calculations and boundary checks for the dim dimensions of an individual. Assuming that the time complexity of each mutation operation is $O\left(p\times \dim\right)$ (where *p* is a constant related to the mutation strategy), the time complexity of mutating N individuals is $O\left(N\times p\times \dim\right)$. The opposition-learning operation also conducts calculations and boundary control for N individuals, and its time complexity is $O\left(N\times \dim\right)$. Adding the original individual update, boundary control, global optimal solution update, and population-sorting operations of t PO, the time complexity of each iteration is $O\left(N\times m\times \dim+N\times \dim+N+N\log N+N\times p\times \dim+N\times \dim\right)$, and the time complexity of the entire iteration stage is $O\left(Max\_iter\times \left(N\times m\times \dim+N\times \dim+N+N\log N+N\times p\times \dim+N\times \dim\right)\right)$.

Combining the initialization and iteration stages, the time complexity of CGBPO is $O\left(N\times \dim+N\times k+N\log N+Max\_iter\times \left(N\times m\times \dim+N\times \dim+N+N\log N+N\times p\times \dim+N\times \dim\right)\right)$. When N, dim, and Max_iterare large, by ignoring the lower-order terms, the main time complexity can be approximated as $O\left(Max\_iter\times N\times \dim\right)$.

#### 3.6.3. Comparison of the Time Complexities of the Two Algorithms

As can be seen from the above analysis, after ignoring the lower-order terms, the main time complexities of PO and CGBPO are $O\left(Max\_iter\times N\times \dim\right)$ approximately. This means that in large-scale problems, when N, Max_iter, and dim increase, the growth trends in the calculation times of the two algorithms are basically the same.

The CGBPO algorithm adds mutation and opposition-based learning operations during the iteration process. There are additional terms related to mutation and opposition-based learning, namely Term $O\left(Max\_iter\times N\times p\times \dim\right)$ and Term $O\left(Max\_iter\times N\times \dim\right)$, in its time–complexity expression. In an actual operation, if the mutation and opposition-based learning operations are computationally complex, the time for each iteration of CGBPO will be longer than that of PO. However, this may also bring better optimization effects, helping the algorithm converge to a better solution more quickly.

#### 3.6.4. Experimental and Comparative Analyses of the Time

Table 1 presents the running times of the CGBPO and PO algorithms, along with the ratio of CGBPO’s running time to that of PO. Using the CEC2022 standard dataset and MATLAB simulation software, the test sets the population size at 30 and the maximum number of iterations at 300. Each of the algorithms runs independently 30 times for each test function. Overall, at a low dimension of 30, CGBPO generally takes longer to run than PO, showing lower computational efficiency. But as the dimension rises to 50 and 100, the running-time gap between the two narrows. Different test functions affect the running-time ratio differently. For complex functions, the ratio drops more notably with increasing dimension, indicating CGBPO’s potential in handling high-dimensional complex functions.

## 4. Experimental and Comparative Analyses of the CEC2017 Benchmark Suite

To evaluate CGBPO’s performance, the CEC2017 and CEC2022 benchmarks were selected. Using MATLAB simulation software, nine algorithms were tested and compared: Parrot Optimization (PO), Harris Hawks Optimization (HHO) [16], Antlion Optimizer (AO) [36], FOX optimization [37], Beluga Whale Optimization (BWO) [38], GOOSE optimization [39], Whale Optimization (WOA) [40], CMA-ES [41], and CGBPO. The population size was set to 30, the maximum iterations to 300, and the dimension to 10, with other parameters following the original literature. Each algorithm ran independently 30 times.

**Figure 6:**
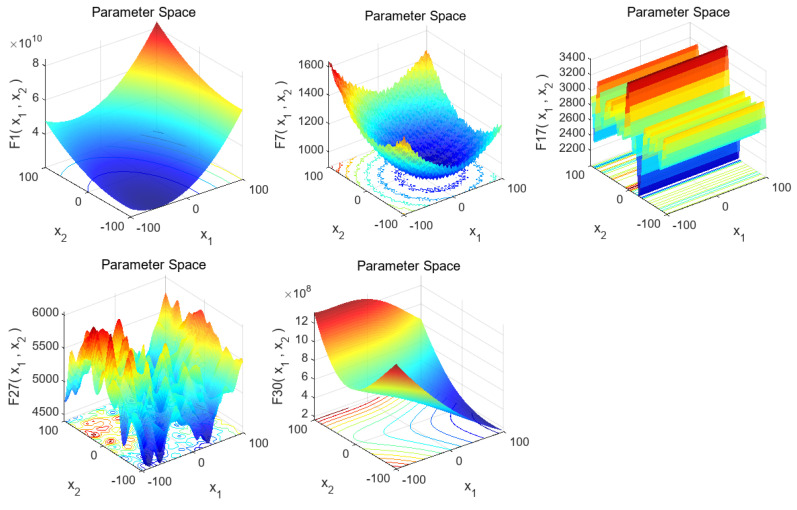
Three-dimensional graphs of some test functions in the CEC2017 benchmark suite.

The CEC2017 benchmark has 29 functions. F1, F3, and F4 are unimodal functions, used to assess global convergence; F5–F11 are simple multi-modal functions for testing the ability to escape local optima; F12–F21 are hybrid functions, F22–F27 are composition functions, and F28–F30 are extended unimodal functions, all for testing algorithms’ handling of complex issues. Some CEC2017 test functions’ 3D graphs are shown in Figure 6.

F1 has an obvious global minimum near the image bottom center, useful for evaluating if an algorithm can find the global optimum. F7 is multimodal with multiple local and a global extreme point, often used to evaluate global search and escaping local optima abilities. F17 has a complex stepped-like structure with multiple local extreme values for evaluating an algorithm’s global-optimum-finding ability for multi-extreme-value functions. F27 is extremely complex multimodal with many local extreme points used to evaluate an algorithm’s global optimum finding and avoiding local optima abilities in complex multimodal scenarios. F30 has a complex surface with multiple local extreme regions for evaluating an algorithm’s performance in complex function environments, including global search, convergence speed, and avoiding local optima.

### 4.1. Statistical Results of Comparative Tests

Comparing CGBPO with eight other algorithms on CEC2017, Table 2, Table 3 and Table 4 reveal its significant performance edge. On unimodal functions, CGBPO has notable advantages on F1, F3, and F4, though CMA-ES excels best on them. For simple multimodal functions, CGBPO performs optimally on F5, F6, F8–F10. In hybrid functions, CGBPO outperforms others on F16, F17, F20, and F21; CMA-ES tops F12–F15, F18, and F19, with CGBPO ranking second. For composition functions, CGBPO leads F22, F27, and F28 and is competitive on others, while CMA-ES performs best overall.

Data shows that CGBPO performs well on various test functions, simple or complex. Analyzing multiple functions, CGBPO generally surpasses PO and some other algorithms (like HHO, AO, BWO) in convergence speed, solution accuracy, and stability. This proves CGBPO’s comprehensive performance advantage and its ability to offer better solutions for practical engineering problems.

### 4.2. Algorithm Convergence Curve on CEC2017

**Figure 7:**
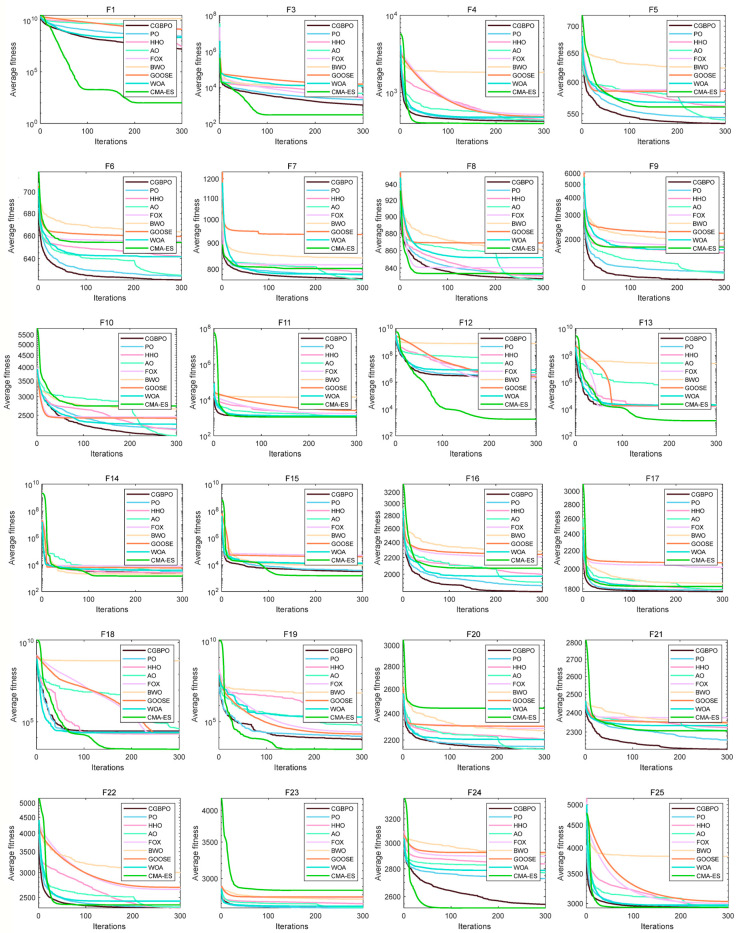

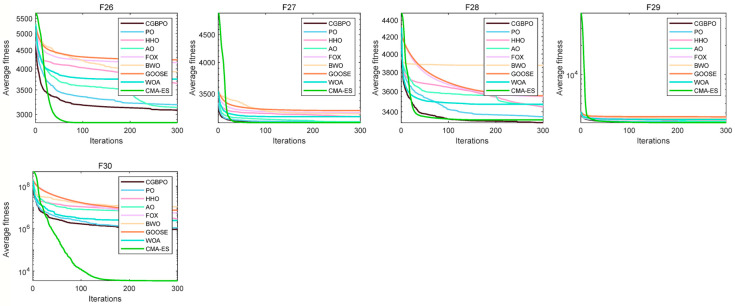
Convergence curves of the proposed and compared functions on CEC2017.

Figure 7 displays the comparative convergence curves of CGBPO and eight other algorithms for functions F1–F30. In most functions’ convergence profiles, the CGBPO curve trends sharply downward. For unimodal functions, CGBPO’s downward trend, convergence speed, and final fitness value rank second only to CMA-ES.

On simple multimodal functions like F6, F8, F9, and F10, CGBPO’s curve has a clear downward trend with a fast decline rate, reaching a low fitness value early in iteration and having a lower final convergence fitness than most other algorithms. For F7, while other algorithms’ curves fluctuate greatly, CGBPO remains stable and achieves better convergence.

On hybrid functions, such as F16, F17, F20, and F21, CGBPO’s curve drops rapidly and reaches a low fitness value early, outperforming others. On F12–F15, F18, and F19, CMA-ES performs best and CGBPO ranks second, still ahead of other algorithms.

For composition functions like F22, 24, 25, F27, and F28, CGBPO’s curve drops quickly and has a low final fitness value for F22, F27, and F28, showing the best performance. For other functions, CGBPO is also competitive. Despite CMA-ES performing better on some functions, overall, CGBPO shows excellent or strong performance across various functions and has a comprehensive performance advantage.

### 4.3. Algorithm Box Plot on CEC2017

**Figure 8:**
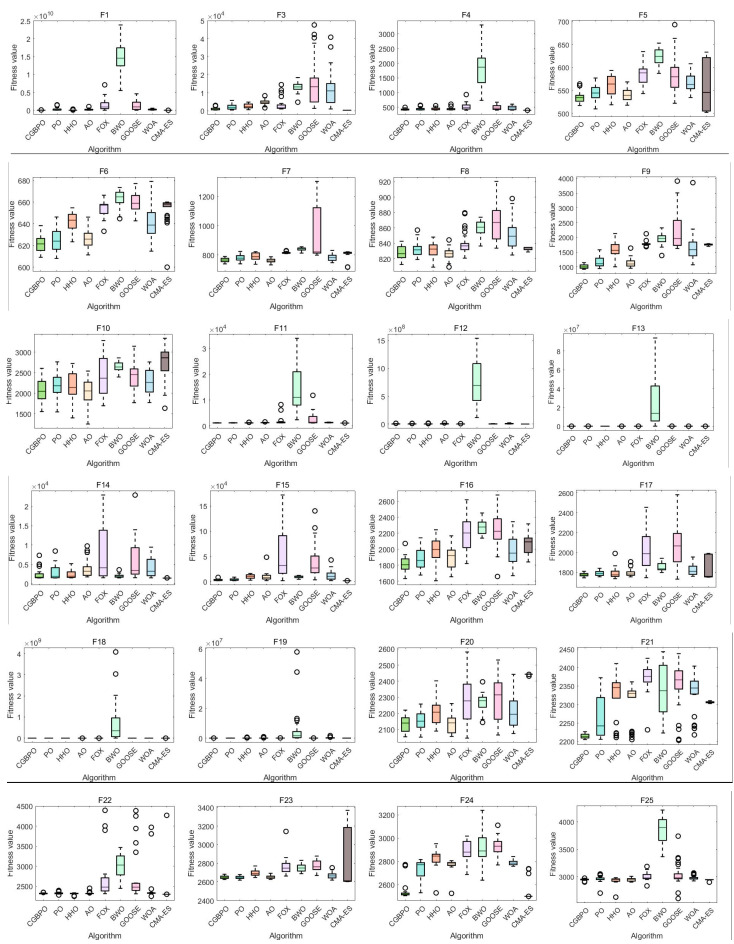

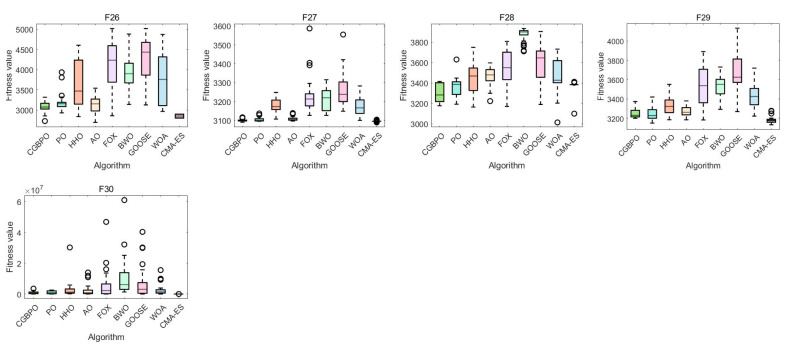
Box plots of functions on CEC2017.

Figure 8 shows box plots of CGBPO and other algorithms. In box plots of multiple functions, CGBPO’s box and whisker lengths indicate data dispersion. Its constituent elements include:The rectangular box in the middle can show the distribution range of the middle 50% of the data; The horizontal line in the middle of the box represents the median of the data, which divides the data into two parts with equal quantities and reflects the central tendency of the data; The line segments extending from the upper and lower ends of the box show the distribution range of the data. The points outside the whiskers are abnormal values that deviate significantly from the overall data.

For unimodal function F1, CGBPO’s box plot is at the lowest, with the smallest median fitness value, converging to a better solution accurately. For F3, CMA-ES’s box plot is lowest, with CGBPO second. For F4, CGBPO performs well with a low median fitness and high accuracy. CGBPO’s box plots for F1 and F4 are short, showing strong stability; for F3, CMA-ES is relatively stable.

For simple multimodal functions like F6, F8, F9, and F10, CGBPO’s box plots are low, with small median fitness values, converging to better solutions accurately. It also excels in F7, more accurate than most algorithms. These box plots are mostly short, indicating good stability.

For hybrid functions such as F16, F17, F20, and F21, CGBPO’s box plots are low, with high accuracy in converged solutions. For F12–F15, F18, and F19, CMA-ES performs best, CGBPO second, still having an accuracy advantage. For good-performing functions of CGBPO, box plots are short with good stability; for CMA-ES-dominated functions, CGBPO also has good stability.

For composition functions like F22, F27, and F28, CGBPO’s box plots are at the lowest, with the highest convergence accuracy. For other functions, CGBPO is competitive, converging to good solutions. For advantageous functions, its box plots are short, indicating good stability; for others, its overall stability is acceptable and comparable to other algorithms.

In general, CGBPO shows excellent comprehensive performance for different functions, having advantages in both convergence accuracy and stability for many functions.

### 4.4. Wilcoxon’s Rank-Sum Test on CEC2017

Table 5 compares the *p*-values of CGBPO and swarm intelligence algorithms via Wilcoxon’s rank-sum test [42] on the CEC2017 benchmark. For functions like F3, F4, F9, F16, F21, and F24, CGBPO’s *p*-values compared to the other eight algorithms are all much less than 0.05, showing its performance significantly surpasses theirs.

In some functions, the *p*-values between CGBPO and specific algorithms exceed 0.05, meaning no significant performance difference. For example, on function F13, CGBPO’s *p*-values relative to PO, HHO, AO, and WOA are 8.53 × 10^−1^, 4.20 × 10^−1^, 1.41 × 10^−1^, and 2.84 × 10^−1^, respectively, indicating similar performance.

Overall, CGBPO performs excellently in most function tests, showing significant differences from and often outperforming other algorithms. Even when its performance is like some algorithms for certain functions, its advantages are still evident, proving its strong competitiveness in solving CEC2017 benchmark problems.

### 4.5. Radar Chart and Average Ranking Chart

**Figure 9:**
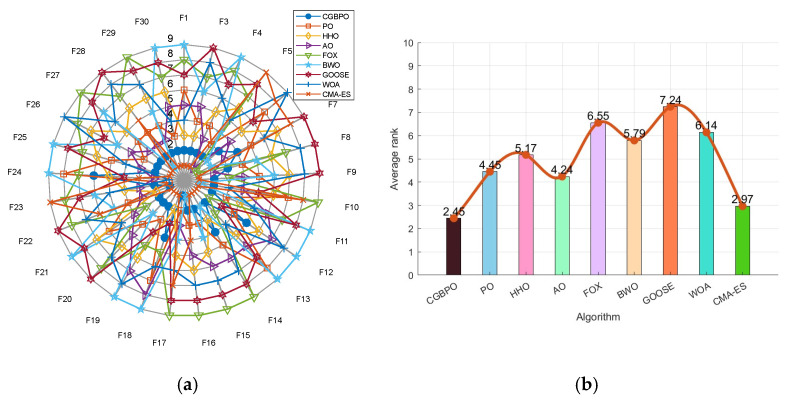
Radar chart (**a**) and ranking chart (**b**) for functions in CEC2017.

Figure 9 show the radar chart and average ranking chart of CGBPO and eight other intelligent algorithms on CEC2017. Notably, CGBPO’s radar chart has the least area fluctuation. It ranked first for two functions and second for twenty functions, indicating highly stable results across multiple runs, unaffected by random factors.

Significantly, CGBPO had the lowest average fitness value and topped the ranking. This shows that in the comprehensive test, CGBPO obtained better solutions and outperformed the other eight algorithms in convergence performance.

### 4.6. Analysis of High-Dimensional Function Tests

To confirm CGBPO’s superiority in handling high-dimensional complex problems, the dimension was set to 100 with other parameters unchanged. High-dimensional function test results in Table 6, Table 7 and Table 8 show that on all high-dimensional unimodal functions, CGBPO has notable advantages and stability in minimum and mean values. On multimodal functions, it significantly outperforms other algorithms. On hybrid and composition functions, it reaches the theoretical optimal value. In some functions’ standard deviation data, it ranks second only to CMA-ES. Overall, CGBPO still excels in high-dimensional complex problems and offers better solutions.

## 5. Experimental and Comparative Analyses on the CEC2022 Benchmark Suite

**Figure 10:**
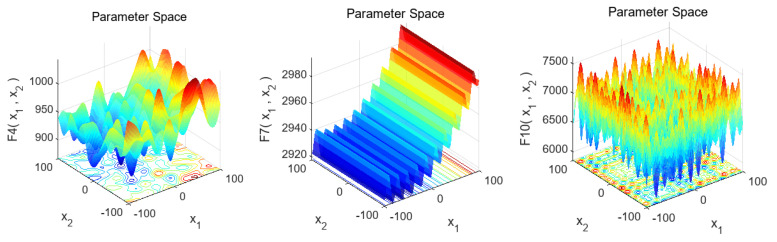
Three-dimensional graphs of some test functions in CEC2022.

The CEC2022 benchmark suite has 12 single-objective test functions with boundary constraints, categorized as follows: F1 is a unimodal function for evaluating convergence speed and accuracy; F2–F5 are multimodal functions with multiple local optima, testing global search capabilities; F6-F8 are hybrid functions for comprehensively assessing algorithm performance under complex conditions; F9–F12 are composite functions for testing the ability to handle complex tasks. Some CEC2022 test functions’ 3D graphs are shown in Figure 10.

F4 has a complex multimodal shape with many local extreme points used to evaluate optimization algorithms’ performance in multimodal environments, like global search and avoiding local optima. F7 has a stepped distribution with multiple levels and potential multiple local extreme values used to evaluate algorithms’ ability to find the global optimum for complex multi-extreme-value functions. F10 has a highly complex multimodal shape and numerous local extreme points, making its optimization difficult, and can evaluate algorithms’ performance in complex multimodal scenarios, such as finding the global optimum and escaping local optima.

### 5.1. Statistical Results of Algorithm Tests on CEC2022

Table 9 shows the min, std, and avg data of CGBPO and eight other algorithms for 12 test functions. On unimodal function F1, CMA-ES performs best. CGBPO’s minimum and average values rank second only to CMA-ES and are much better than others, indicating high convergence accuracy on unimodal functions.

On multimodal function F3, CGBPO has the best minimum value and small standard deviation, showing strong stability. On F4, algorithms’ minimum values are close, and CGBPO’s has an edge with a reasonable standard deviation. On F5, CGBPO’s minimum value is better than most, except CMA-ES, proving good comprehensive performance on multimodal functions.

On hybrid function F6, CMA-ES has the best minimum value, and CGBPO ranks second but has a large standard deviation, resulting in poor stability. On F7, CGBPO has the best minimum value and small standard deviation, showing good stability. On F8, CMA-ES has the best minimum value, CGBPO ranks third with a small standard deviation, indicating strong stability. CGBPO is competitive overall despite performance fluctuations on hybrid functions.

On composition function F9, CMA-ES has the best minimum value, CGBPO ranks second, close to CMA-ES, and has a small standard deviation. On F10, CGBPO has the best minimum value and extremely small standard deviation, showing excellent stability. On F11, CMA-ES has the best minimum value, CGBPO ranks third with a small standard deviation. On F12, CMA-ES has the best minimum value, and CGBPO ranks second with a small standard deviation, indicating good comprehensive performance on composition functions.

In general, CGBPO shows high convergence accuracy, strong stability, and good global convergence on various functions. Its comprehensive performance is remarkable among many algorithms. Despite some flaws in individual functions, its overall performance is good, with obvious advantages on unimodal, multimodal, and composition functions.

### 5.2. Algorithm Convergence Curve on CEC2022

**Figure 11:**
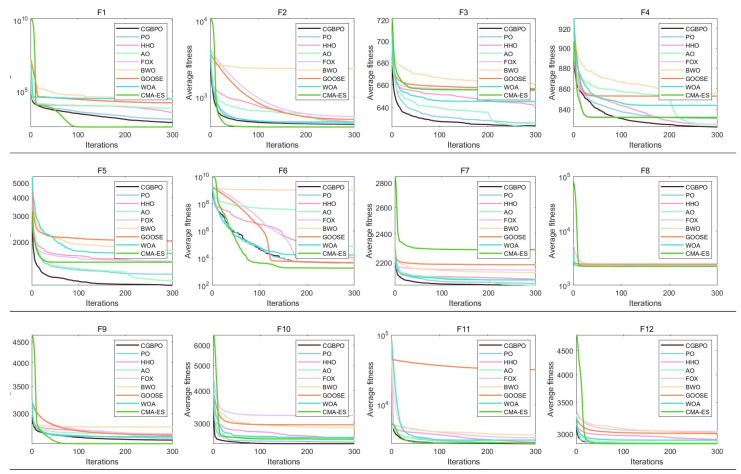
Convergence curves of functions on CEC2022.

Figure 11 displays the convergence graphs of CGBPO and other algorithms for functions F1–F12. For unimodal function F1, CMA-ES’s curve drops fastest with the lowest final average fitness, performing best. CGBPO’s curve also drops quickly, with its final average fitness ranking second only to CMA-ES and better than others, showing good convergence speed and accuracy.

For multimodal functions, in F3, CGBPO’s curve starts low with a clear downward trend and the lowest final average fitness, performing best. In F4, algorithms’ curves are close initially, but CGBPO’s has a better downward trend later with the optimal average fitness. In F5, CGBPO’s curve drops rapidly and has the lowest final average fitness, indicating good convergence speed and accuracy.

For hybrid functions, in F6, CMA-ES’s curve has an obvious downward trend later with the lowest final average fitness, while CGBPO’s performs well early but is overtaken later. In F7, CGBPO’s curve has the lowest final average fitness. In F8, CMA-ES’s curve has the lowest final average fitness, and CGBPO’s drop smoothly. CGBPO is competitive on hybrid functions, though less so than on unimodal and multimodal ones.

For composition functions, in F9, CMA-ES and CGBPO’s curves have similar downward trends with close and low final average fitness, CGBPO being slightly worse. In F10, CGBPO’s curve drops rapidly and has the lowest final average fitness, performing best. In F11, algorithms’ curves vary greatly, and CGBPO’s drop fast early. In F12, CMA-ES’s curve has the lowest final average fitness, and CGBPO’s ranks second, showing good overall performance on composition functions.

### 5.3. Algorithm Box Plot on CEC2022

**Figure 12:**
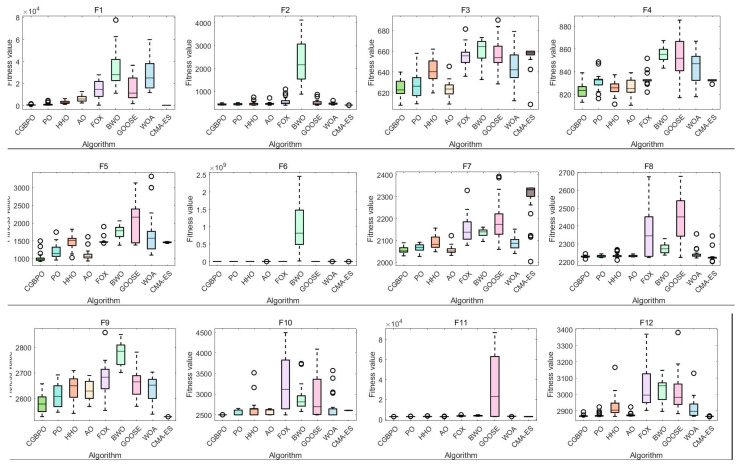
Box plot of functions on CEC2022.

Figure 12 shows box plots of test data distribution. For unimodal function F1, CMA-ES’s box plot is at the lowest, with the smallest fitness median and the best converged solution. CGBPO’s box plot is low with a short box, meaning it converges with a good solution and has small data dispersion, showing good stability.

For multimodal function F3, CGBPO’s box plot is at the lowest, with the smallest fitness median, indicating the best performance and converging to a better solution. Its box is short, showing good stability. For F4, CGBPO’s box plot is at the lowest. Despite outliers, the overall fitness median is small, with a good convergence effect. For F5, CGBPO’s box plot is at the lowest, with a fitness value much lower than most algorithms, showing high convergence accuracy.

For hybrid function F7, CGBPO’s box plot is low, with a small fitness median, good convergence, and small data dispersion, indicating good stability. For F8, CMA-ES’s box plot is at the lowest, performing best, while CGBPO’s is at an intermediate level, competitive but slightly worse.

For composition function F10, CGBPO’s box plot is at the lowest, with the smallest fitness median, the best performance, and a short box for good stability. For F12, CMA-ES’s box plot is at the lowest, and CGBPO’s is the second lowest, with good performance.

In general, CGBPO has obvious advantages on multimodal and composition functions, strong competitiveness on unimodal and hybrid functions, and outstanding comprehensive performance across different functions.

### 5.4. Wilcoxon’s Rank-Sum Test on CEC2022

Table 10 shows the rank-sum test results of the CEC2022 benchmark test. When comparing CGBPO with BWO, FOX, GOOSE, WOA, and CMA-ES, the *p*-values of all 12 functions are below 0.05, indicating that CGBPO significantly outperforms PO and BWO.

For the four functions, the *p*-values of CGBPO relative to AO exceed 0.05, meaning CGBPO and AO have similar performance on these functions.

Overall, CGBPO performs remarkably in most function tests, showing significant differences from and often outperforming other algorithms. Clearly, CGBPO maintains strong competitiveness in solving the CEC2022 benchmark problems.

### 5.5. Radar Chart and Average Ranking Chart

**Figure 13:**
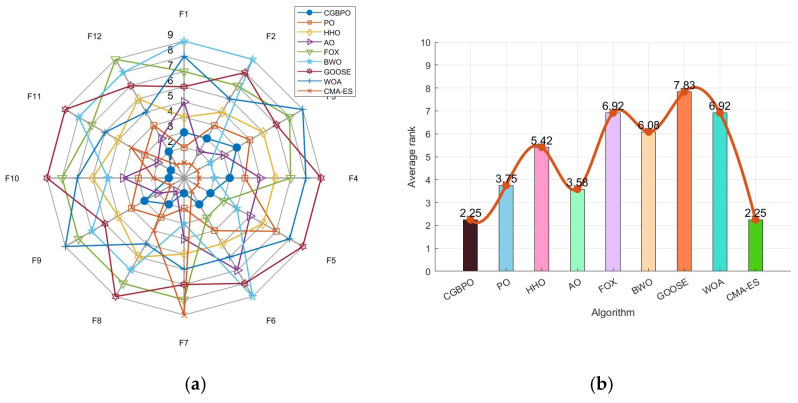
Radar chart (**a**) and ranking chart (**b**) for functions in CEC2022.

Figure 13 shows a radar chart and an average ranking chart comparing CGBPO with seven other algorithms on CEC2022. Notably, CGBPO’s radar chart has the least fluctuations. It ranked first for three functions (including F10) and second for four (like F5), showing remarkable performance and distinct advantages on these functions in Figure 13a.

The CGBPO algorithm had an average ranking of 2.25, tying for first place with CMA-ES in Figure 13b. It demonstrated outstanding comprehensive performance across multiple test functions, outperforming the other seven algorithms overall.

## 6. Application in Engineering Problems

To verify CGBPO’s performance in handling complex engineering problems, tests were conducted on the design optimization of industrial refrigeration systems [43] and the optimization of Himmel Blau’s function [44]. Then, the optimization results were compared and analyzed with those of the other seven algorithms mentioned above.

### 6.1. Optimization Results for Industrial Refrigeration Systems

With the continuous depletion of basic energy resources, energy conservation and emissions reduction have become a key concern across all industries recently. Industrial refrigeration systems consume a large amount of energy in enterprises. The design optimization of such systems seeks to strike a fine balance among performance, cost, and efficiency. This design issue involves 14 design variables and 15 constraint conditions in total. The mathematical model thereof is presented as follows:

Design variables:

(13) $$\vec{x}=\left[x_{1}x_{2}x_{3}x_{4}x_{5}x_{6}x_{7}x_{8}x_{9}x_{10}x_{11}x_{12}x_{13}x_{14}\right]$$

Objective function:

(14) $$\begin{array}{l} f\left(\vec{x}\right)=63098.88x_{2}x_{4}x_{12}+5441.5x_{2}^{2}x_{12}+115055.5x_{2}^{1.664}x_{6}+6172.27x_{2}^{2}x_{6}\\ +63098.88x_{1}x_{3}x_{11}+5441.5x_{1}^{2}x_{11}+115055.5x_{1}^{1.664}x_{5}+6171.27x_{1}^{2}x_{5}\\ +140.53x_{1}x_{11}+281.29x_{3}x_{11}+70.26x_{1}^{2}+281.29x_{1}x_{3}+281.29x_{3}^{2}\\ +14437x_{8}^{1.8812}x_{12}^{0.3424}x_{10}x_{14}^{-1}x_{7}x_{9}^{-1}+20470x_{7}^{2.893}x_{11}^{0.316}x_{1}^{2} \end{array}$$

Constraint conditions:

(15) $$\begin{array}{l} g_{1}\left(\vec{x}\right)=1.524x_{7}^{-1}\leq 1\\ g_{2}\left(\vec{x}\right)=1.524x_{8}^{-1}\leq 1\\ g_{3}\left(\vec{x}\right)=0.07789x_{1}-2x_{7}^{-3}x_{9}-1\leq 0\\ g_{4}\left(\vec{x}\right)=7.05305x_{9}^{-1}x_{1}^{2}x_{10}x_{8}^{-1}x_{2}^{-1}x_{14}^{-1}-1\leq 0\\ g_{5}\left(\vec{x}\right)=0.0833x_{13}^{-1}x_{14}-1\leq 0\\ g_{6}\left(\vec{x}\right)=47.136x_{2}^{0.333}x_{10}^{-1}x_{12}-1.333x_{8}x_{13}^{2.1195}+62.08x_{13}^{2.1195}x_{12}^{-1}x_{8}^{0.2}x_{10}^{-1}-1\leq 0\\ g_{7}\left(\vec{x}\right)=0.04771x_{10}x_{8}^{1.8812}x_{12}^{0.3424}-1\leq 0\\ g_{8}\left(\vec{x}\right)=0.0488x_{9}x_{7}^{1.893}x_{11}^{0.316}-1\leq 0\\ g_{9}\left(\vec{x}\right)=0.0099x_{1}x_{3}^{-1}-1\leq 0\\ g_{10}\left(\vec{x}\right)=0.0193x_{2}x_{4}^{-1}-1\leq 0\\ g_{11}\left(\vec{x}\right)=0.0298x_{1}x_{5}^{-1}-1\leq 0\\ g_{12}\left(\vec{x}\right)=0.056x_{2}x_{6}^{-1}-1\leq 0\\ g_{13}\left(\vec{x}\right)=2x_{9}^{-1}-1\leq 0\\ g_{14}\left(\vec{x}\right)=2x_{10}^{-1}-1\leq 0\\ g_{15}\left(\vec{x}\right)=x_{12}x_{11}^{-1}-1\leq 0 \end{array}$$

Range of values:

(16) $$0.001\leq x_{i}\leq 5,i=1,\ldots ,14$$

Table 11 compares the optimal results of the CGBPO algorithm with those of other methods. Clearly, CGBPO achieved the minimum optimized value of 5.32 × 10^−1^, showing its ability to meet stricter precision requirements. Also, CGBPO’s average value and standard deviation were lower than those of the other eight algorithms.

**Figure 14:**
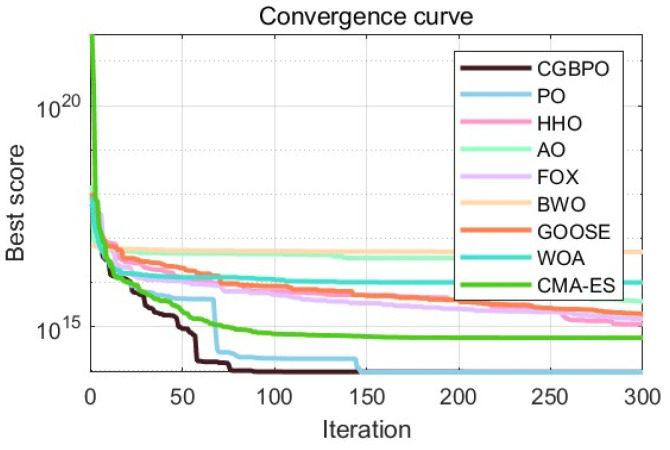
Convergence curves regarding the design optimization problem for industrial refrigeration systems.

**Figure 15:**
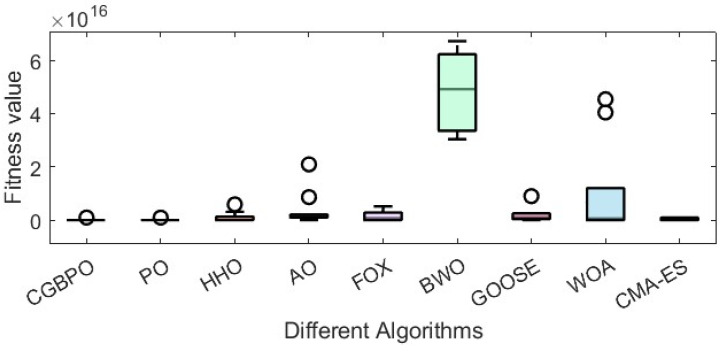
Box plots regarding the design optimization problem for industrial refrigeration systems.

By comparing the convergence curves in Figure 14 and the box plots in Figure 15, it is evident that CGBPO was the most stable and converged fastest in the later iteration stage. This means CGBPO can optimize the refrigeration system design more quickly, reducing the system adjustment time while ensuring system performance.

In conclusion, CGBPO showed superiority and suitability in solving the design optimization problem of industrial refrigeration systems.

### 6.2. Optimization of Himmel Blau’s Function

Himmel Blau’s function is a commonly used multimodal function for evaluating optimization algorithm performance. It is mainly applied to analyze non-linear constrained optimization problems. Notably, this function has six non-linear constraints, involves five variables, and its mathematical expression is as follows:

Minimize

(17) $$f\left(\vec{x}\right)=5.3578547x_{3}^{2}+0.8356891x_{1}x_{5}+37.293239x_{1}-40792.141$$

subject to

(18) $$\begin{array}{l} g_{1}\left(\vec{x}\right)=-G1\leq 0,\\ g_{2}\left(\vec{x}\right)=G1-92\leq 0,\\ g_{3}\left(\vec{x}\right)=90-G2\leq 0,\\ g_{4}\left(\vec{x}\right)=G2-110\leq 0,\\ g_{5}\left(\vec{x}\right)=20-G3\leq 0,\\ g_{6}\left(\vec{x}\right)=G3-25\leq 0, \end{array}$$

where

(19) $$\begin{array}{l} G1=85.334407+0.0056858x_{2}x_{5}+0.0006262x_{1}x_{4}-0.0022053x_{3}x_{5},\\ G2=80.51249+0.0071317x_{2}x_{5}+0.0029955x_{1}x_{2}+0.0021813x_{3}^{2},\\ G3=9.300961+0.0047026x_{3}x_{5}+0.00125447x_{1}x_{3}+0.0019085x_{3}x_{4}, \end{array}$$

with the bounds

(20) $$\begin{array}{l} 78\leq x_{1}\leq 102,\\ 33\leq x_{2}\leq 45,\\ 27\leq x_{3}\leq 45,\\ 27\leq x_{4}\leq 45,\\ 27\leq x_{5}\leq 45. \end{array}$$

**Figure 16:**
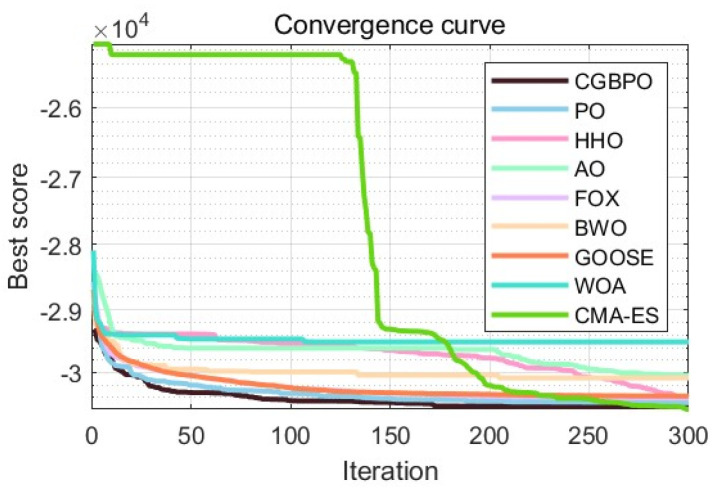
Convergence curves for Himmel Blau’s function optimization problem.

**Figure 17:**
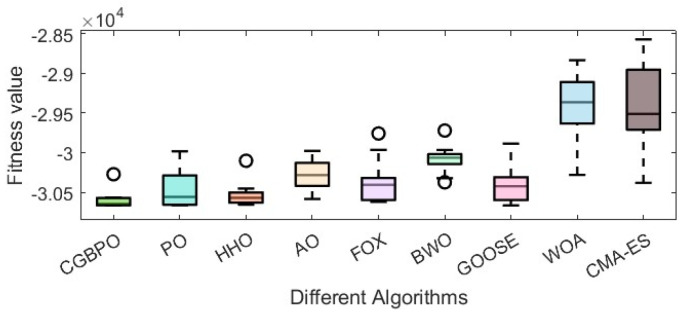
Box plots for Himmel Blau’s function optimization problem.

As Table 12 shows, CGBPO achieves a minimum value of −30,665.4, the lowest among compared algorithms. Regarding std and avg values, CGBPO outperforms its counterparts. From Figure 16 and Figure 17, CGBPO has the fastest convergence speed when optimizing functions, obtaining the optimal solution earliest, showing the best stability, and having the highest solution accuracy.

These results clearly prove that CGBPO excelled in solving Himmel Blau’s function optimization problem. It not only had an edge in finding the optimal solution but also surpassed other algorithms in stability and overall performance, making it highly competitive.

## 7. Application of the CGBPO Algorithm in Indoor Visible Light Positioning

The CGBPO algorithm is applied to indoor visible light positioning [45]. In the indoor wireless visible-light transmission model, Light-Emitting Diodes (LEDs) are signal sources for data transmission, and Photo Diodes (PDs) are receivers for data reception to achieve high-precision positioning. A 5 m × 5 m × 6 m positioning model is established. Four LEDs on the ceiling, with coordinates (5, 0, 6), (0, 0, 6), (0, 5, 6), and (5, 5, 6), respectively, are set as signal sources.

To test the positioning error of CGBPO, at a height of 2 m, signal receivers are placed every 0.5 m in the 5 m length and 5 m width directions, creating 121 test points. A positioning-simulation experiment is conducted using MATLAB, and the experiment’s relevant parameters are set as shown in Table 13.

**Figure 18:**
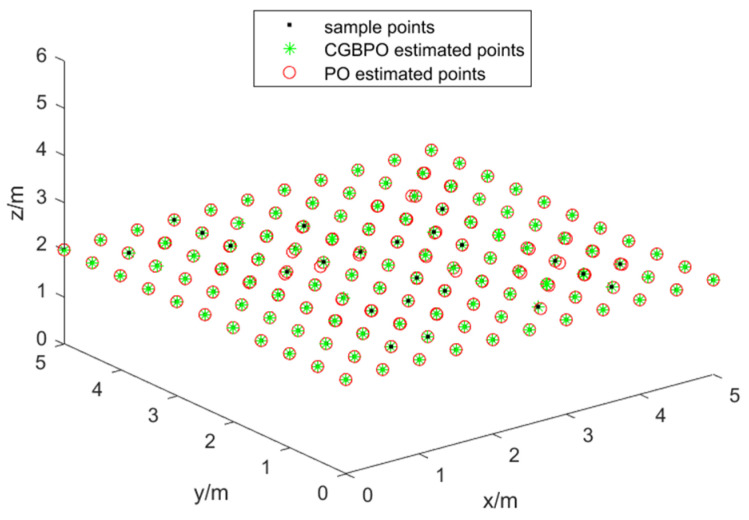
Distribution diagram of actual location.

Figure 18 displays the distribution of the actual positions of PDs and the estimated positions from PO. The CGBPO algorithm’s estimated positions are nearer to the actual PD positions. Moreover, CGBPO has the optimal coverage, which reaches 100%.

**Figure 19:**
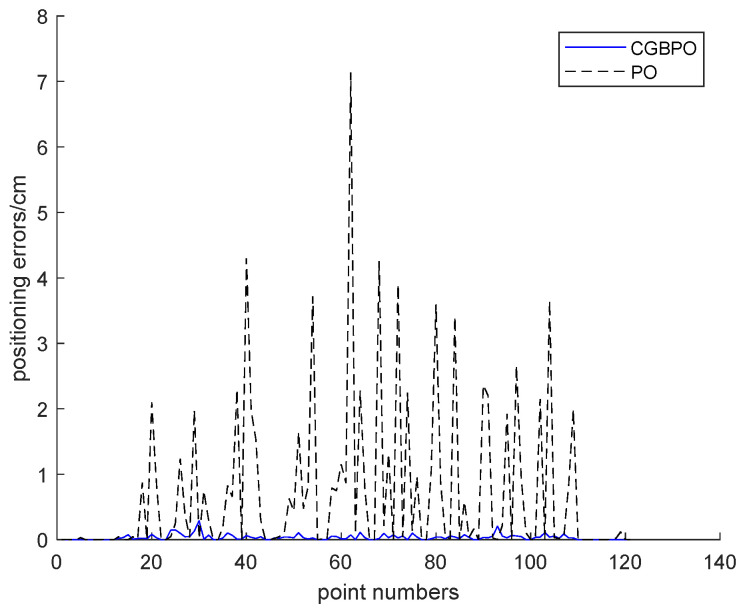
Curve of estimated position error.

Figure 19 presents the error curves of the estimated positions for the two algorithms. Clearly, among the 121 test points, CGBPO has the smallest error in estimated positions, while PO shows relatively larger errors at each test point.

**Figure 20:**
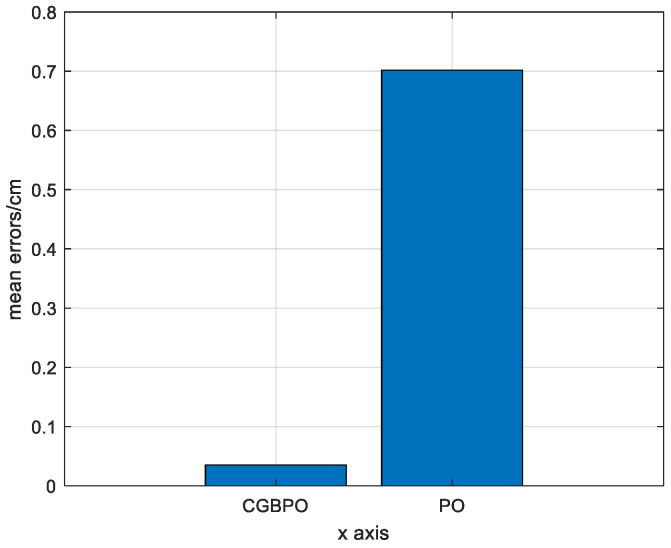
Comparison of average errors of estimated positions.

Figure 20 is a bar chart of the average errors of the estimated positions for the two algorithms. By comparison, the average error of PO’s estimated positions is about 0.0070 cm, while that of CGBPO is about 0.00034807 cm. The CGBPO algorithm shows the most stable positioning performance.

## 8. Conclusions

To overcome the drawbacks of the PO algorithm, like becoming stuck in local optima and slow convergence for complex problems, this study proposed the CGBPO algorithm, which has improved performance and applicability. CGBPO uses chaotic logistic mapping for initialization to increase population diversity, applies Gaussian mutation to update individual positions to avoid premature local convergence, and incorporates the barycenter opposite learning approach to generate opposite solutions and boost global search ability. Simulation experiments on CEC2017 and CEC2022 benchmarks, comparing them with eight intelligent optimization algorithms, and two complex engineering problems showed that CGBPO can improve solution accuracy and convergence speed, balancing global and local search. In industrial refrigeration system design optimization and Himmel Blau’s function optimization, CGBPO achieved the highest accuracy, shortest optimization time, and best stability. In indoor visible light positioning, CGBPO’s estimated positions were closer to actual PD positions, with the best coverage and smallest average error (about 0.00034807 cm) compared to PO.

Future research will explore integrating CGBPO with other advanced methods like the MAMGD optimization method, aiming to further improve convergence speed and training-result accuracy [46]. Combining CGBPO with the differential evolution (DE [47]) algorithm and implementing multi-objective optimization (e.g., extending to NSGA-II [48]) will also be considered to enhance its performance, expand applicability, and meet the increasing needs for complex optimization.

## Figures and Tables

**Table 1:** Test results of the Avg_Time.

Function	Avg_Time of Dim 30			Avg_Time of Dim 50			Avg_Time of Dim 100		
	CGBPO	PO	Ratio	CGBPO	PO	Ratio	CGBPO	PO	Ratio
F1	3.15	0.94	3.35	6.08	2.35	2.59	6.42	3.08	2.08
F3	3.20	0.93	3.44	2.73	1.13	2.42	4.96	3.05	1.63
F4	2.87	0.89	3.22	2.75	1.15	2.39	5.00	3.05	1.64
F5	3.63	1.43	2.54	3.48	1.70	2.05	6.16	4.00	1.54
F6	4.71	2.04	2.31	5.57	2.99	1.86	9.37	6.72	1.39
F7	5.13	2.36	2.17	3.35	1.67	2.00	6.32	4.13	1.53
F8	4.41	1.40	3.15	3.29	1.61	2.04	6.36	4.16	1.53
F9	4.12	1.52	2.71	3.35	1.70	1.97	6.27	4.10	1.53
F10	5.31	1.65	3.22	3.73	1.97	1.89	7.09	4.69	1.51
F11	3.48	1.14	3.05	2.93	1.31	2.24	5.50	3.50	1.57
F12	3.62	1.43	2.53	3.29	1.65	1.99	6.24	4.11	1.52
F13	4.35	1.28	3.40	2.97	1.37	2.17	5.61	3.58	1.57
F14	3.96	1.68	2.36	3.57	1.94	1.84	7.04	4.73	1.49
F15	3.85	1.14	3.38	2.84	1.30	2.18	5.45	3.42	1.59
F16	3.39	1.51	2.25	3.17	1.49	2.13	5.94	3.83	1.55
F17	5.66	2.25	2.52	4.49	2.51	1.79	8.33	5.70	1.46
F18	3.63	1.39	2.61	3.12	1.50	2.08	6.05	3.87	1.56
F19	13.45	8.79	1.53	12.73	9.09	1.40	23.38	18.31	1.28
F20	6.17	3.96	1.56	4.92	2.75	1.79	8.68	6.06	1.43
F21	6.74	3.80	1.77	6.44	3.68	1.75	17.12	13.37	1.28
F22	8.19	4.47	1.83	7.49	4.24	1.77	19.64	14.90	1.32
F23	9.22	5.12	1.80	7.95	4.83	1.65	17.94	13.09	1.37
F24	9.23	5.05	1.83	9.21	6.25	1.47	19.36	14.26	1.36
F25	10.37	5.60	1.85	8.07	5.11	1.58	18.70	13.61	1.37
F26	15.90	8.96	1.77	10.00	6.88	1.45	25.67	21.37	1.20
F27	16.42	9.76	1.68	10.13	6.87	1.47	35.19	26.10	1.35
F28	13.24	8.01	1.65	9.47	6.58	1.44	31.88	23.14	1.38
F29	12.76	6.99	1.83	7.94	5.05	1.57	21.53	15.00	1.44
F30	24.97	7.94	3.15	16.12	12.40	1.30	41.24	31.38	1.31

**Table 2:** Test results on CEC2017 (unimodal and simple multi-modal functions).

Function	Criteria	CGBPO	PO	HHO	AO	FOX	BWO	GOOSE	WOA	CMA-ES
F1	min	293,165.4	5,337,319.1	1,135,557.7	2,275,979.9	763,179.6	551,394,6763.3	3,257,262.2	23,872,113.1	100.0
	std	22,384,116.0	371500492.3	63,239,782.4	22,832,8804.7	155,907,6402.5	448,458,6416.4	1,376,810,745.0	166,681,599.3	0.0
	avg	16,201,211.4	287168876.8	27,271,828.4	21,085,0323.9	146,183,6693.3	145,394,67508.3	1,344,318,195.5	210,187,164.4	100.0
F3	min	398.4	405.7	587.9	1628.6	1031.9	4773.8	1340.2	1014.8	300.0
	std	740.6	1466.2	1267.1	1475.3	3369.6	2671.3	12,634.1	9712.1	0.0
	avg	1049.8	2090.4	2588.0	4694.3	3173.4	13,287.7	15,291.4	12,091.7	300.0
F4	min	400.0	407.6	405.5	407.9	401.9	737.8	404.0	404.6	400.0
	std	25.3	43.6	43.6	42.1	111.5	634.9	77.2	60.7	0.0
	avg	428.0	449.8	450.4	442.3	522.9	1831.9	493.8	483.2	400.0
F5	min	501.1	509.6	518.6	517.6	542.8	587.0	521.9	534.4	502.0
	std	13.2	15.2	21.5	13.1	24.0	18.0	37.5	18.1	56.7
	avg	535.5	544.2	561.4	540.8	587.6	623.7	585.2	567.8	560.3
F6	min	600.0	608.0	623.4	611.3	633.1	644.8	642.8	614.8	600.0
	std	7.3	10.6	8.7	8.2	7.7	7.5	9.0	16.1	11.8
	avg	621.9	625.2	641.8	625.8	655.2	663.3	659.3	641.6	654.0
F7	min	719.4	739.9	737.2	732.7	809.4	813.2	799.2	747.6	717.3
	std	14.4	20.0	22.8	12.8	5.7	11.1	169.1	21.1	33.8
	avg	766.0	779.2	790.2	761.7	815.1	840.6	934.3	781.7	801.7
F8	min	812.6	819.2	809.2	809.4	820.9	836.6	833.8	824.9	828.9
	std	1.7	8.7	9.2	7.4	14.1	9.2	23.8	19.1	1.8
	avg	827.9	832.6	830.9	826.7	840.4	860.8	868.7	851.8	833.8
F9	min	916.1	943.6	1000.9	951.1	1683.1	1383.2	1619.7	1069.4	1694.6
	std	23.4	159.3	243.2	147.7	73.9	186.4	593.6	506.4	24.5
	avg	1013.2	1160.2	1586.0	1135.5	1781.3	1952.1	2201.1	1677.2	1752.9
F10	min	1254.1	1546.8	1401.5	1551.6	1696.4	2394.9	1771.5	1774.3	1635.6
	std	164.7	326.8	324.9	314.0	486.7	222.3	319.2	285.0	420.6
	avg	2043.6	2188.0	2166.7	2043.7	2453.2	2657.4	2430.0	2290.4	2735.5
F11	min	1115.3	1109.9	1114.2	1142.6	1150.0	2394.7	1142.6	1161.6	1102.0
	std	39.3	48.9	87.9	105.5	1526.1	8928.6	2261.3	91.7	13.9
	avg	1176.0	1204.4	1207.0	1289.3	1736.8	14,496.2	2607.8	1311.5	1118.6

**Table 3:** Test results on CEC2017 (hybrid functions).

Function	Criteria	CGBPO	PO	HHO	AO	FOX	BWO	GOOSE	WOA	CMA-ES
F12	min	23,166.3	19,048.1	15,396.3	58,156.2	44,296.1	11,496,4067.9	48,982.3	118,189.1	1318.6
	std	3,563,572.6	3,293,097.5	4,722,385.7	5,495,619.8	2,113,319.1	40,180,5763.1	2,541,101.5	6,087,458.8	211.3
	avg	2,671,403.5	2,825,986.4	3,446,835.3	5,087,353.4	1,773,596.2	74,002,0574.9	2,492,890.0	7,943,418.8	1729.3
F13	min	2224.5	1834.7	2030.8	2654.2	2951.6	38,485.7	1792.7	2069.7	1302.0
	std	12,101.8	18,023.8	14,042.9	15,445.9	13,066.4	24,838,681.3	17,417.9	13,474.7	63.0
	avg	15,770.3	18,663.8	15,065.5	20,517.2	15,470.1	24,204,221.3	17,116.0	19,114.9	1330.9
F14	min	1479.7	1480.8	1514.1	1756.8	1526.0	1499.3	1546.2	1510.8	1429.0
	std	1473.5	1808.1	1185.1	2173.7	7015.7	454.6	5071.0	2259.5	21.0
	avg	2425.8	2912.4	2531.4	3790.6	7838.9	2020.3	5854.3	3953.5	1452.5
F15	min	1680.5	1651.0	2668.4	1809.3	1686.9	4393.6	3325.8	1790.6	1501.5
	std	1491.4	1789.2	4001.2	8331.7	49,345.3	2262.1	33,132.5	10,028.8	28.1
	avg	3205.1	3764.8	9849.0	9654.0	50,508.8	9098.2	39,617.9	12,864.2	1531.9
F16	min	1631.8	1676.6	1604.9	1655.0	1820.7	2139.3	1658.6	1668.9	1840.7
	std	92.8	129.0	144.9	139.0	214.1	98.5	212.8	182.1	136.4
	avg	1809.5	1873.1	1994.9	1910.1	2205.4	2279.3	2240.5	1975.6	2069.9
F17	min	1744.3	1754.5	1735.3	1754.1	1745.8	1797.7	1731.0	1760.0	1750.7
	std	15.6	22.7	49.6	34.4	200.2	39.5	199.3	54.0	106.4
	avg	1775.2	1789.1	1790.2	1793.4	2016.3	1852.9	2063.6	1822.7	1824.6
F18	min	3436.7	2370.0	2126.9	3944.3	2343.7	2,411,503.5	2651.3	2410.2	1803.2
	std	13,653.2	16,726.2	12,569.2	26,208.3	14,078.8	97,303,7204.6	13,162.7	14,359.1	32.3
	avg	25,915.7	22,058.7	16,882.7	36,689.5	18,464.6	70,451,5737.9	19,156.0	20,070.8	1849.5
F19	min	1952.9	1998.4	2371.3	2061.4	2332.0	30,596.4	2151.8	2628.1	1903.0
	std	6986.4	11,645.0	120,958.9	179,321.3	22,156.1	12,905,067.3	15,347.9	353,208.3	9.5
	avg	7357.3	12,260.5	52,703.1	61,901.3	22,937.7	6,131,260.1	17,077.6	193,418.1	1914.8
F20	min	2053.2	2055.1	2090.5	2057.6	2046.8	2144.2	2066.1	2075.4	2431.2
	std	2.6	57.4	80.4	57.7	135.9	51.9	139.8	95.2	3.0
	avg	2134.0	2153.9	2205.2	2136.9	2285.1	2269.4	2301.2	2204.9	2442.1
F21	min	2203.0	2205.6	2210.6	2204.9	2231.4	2222.8	2205.0	2217.7	2302.6
	std	1.2	52.5	59.7	50.8	36.5	70.2	67.1	51.3	2.0
	avg	2214.2	2259.8	2322.4	2307.5	2373.3	2344.1	2348.8	2332.8	2306.2

**Table 4:** Test results on CEC2017 (composition functions and extended unimodal functions).

Function	Criteria	CGBPO	PO	HHO	AO	FOX	BWO	GOOSE	WOA	CMA-ES
F22	min	2249.5	2281.3	2247.5	2311.8	2312.1	2446.2	2311.3	2306.9	2300.0
	std	10.5	27.7	17.5	26.1	514.8	304.2	579.1	397.4	359.6
	avg	2314.9	2329.9	2318.8	2325.7	2650.6	3000.3	2692.4	2432.8	2365.7
F23	min	2626.0	2618.7	2648.4	2627.5	2663.3	2688.7	2669.4	2619.3	2603.0
	std	15.8	15.8	31.5	17.3	90.7	35.7	59.3	32.6	311.3
	avg	2649.1	2650.9	2696.0	2653.2	2767.9	2748.3	2774.9	2669.1	2853.9
F24	min	2505.5	2530.8	2529.7	2525.7	2687.5	2638.7	2769.5	2758.1	2500.0
	std	76.8	98.4	76.5	48.7	84.5	137.9	76.0	22.6	68.2
	avg	2545.7	2728.3	2836.9	2771.2	2894.1	2913.2	2926.0	2788.3	2522.3
F25	min	2900.0	2699.3	2625.6	2900.0	2831.1	3363.3	2600.4	2921.4	2898.0
	std	22.3	60.2	62.3	29.4	67.0	259.0	200.9	35.3	14.1
	avg	2941.4	2954.2	2931.6	2946.7	3001.5	3826.5	3031.0	2975.6	2939.5
F26	min	2727.3	2930.6	2836.8	2693.9	2859.2	3139.9	3127.4	2968.1	2800.0
	std	136.4	207.4	600.0	208.0	625.5	413.2	591.5	640.2	50.7
	avg	3077.5	3191.2	3650.9	3136.9	4164.6	3923.0	4234.4	3748.0	2846.7
F27	min	3080.8	3095.5	3107.2	3096.8	3126.9	3126.6	3148.7	3100.4	3089.5
	std	2.3	10.0	35.6	9.7	92.7	58.5	85.1	50.7	2.5
	avg	3090.0	3104.5	3177.1	3108.1	3232.1	3213.4	3258.6	3175.6	3096.1
F28	min	3014.6	3192.6	3164.2	3223.0	3170.5	3710.4	3189.5	3177.1	3100.0
	std	84.7	92.7	156.6	90.3	184.1	64.6	191.7	186.4	125.4
	avg	3297.5	3352.8	3446.6	3469.1	3549.4	3874.4	3557.2	3472.2	3322.9
F29	min	3198.9	3149.5	3184.1	3183.5	3181.9	3292.2	3268.4	3221.6	3131.7
	std	45.1	68.6	100.3	55.8	210.0	97.9	197.4	122.6	37.8
	avg	3249.3	3251.3	3337.4	3272.5	3538.3	3530.6	3663.0	3428.1	3183.8
F30	min	10,510.4	4960.0	137,198.0	12,779.8	6915.9	1,292,883.4	31,211.5	19,250.5	3394.9
	std	841,581.1	879,430.7	5,458,572.3	3,630,522.4	9,404,026.4	12,310,152.4	10,232,108.2	3,397,987.8	62.9
	avg	923,876.5	1,088,041.0	2,857,102.2	2,427,257.0	5,504,069.7	10,697,044.4	7,402,947.6	2,416,955.6	3451.1

**Table 5:** *p*-values obtained from Wilcoxon’s rank-sum test on CEC2017.

Function	PO	HHO	AO	FOX	BWO	GOOSE	WOA	CMA-ES
F1	1.87 × 10^−7^	9.12 × 10^−1^	9.83 × 10^−8^	1.25 × 10^−7^	3.02 × 10^−11^	8.20 × 10^−7^	3.47 × 10^−10^	3.02 × 10^−11^
F3	1.60 × 10^−3^	1.03 × 10^−6^	1.21 × 10^−10^	2.88 × 10^−6^	3.02 × 10^−11^	1.78 × 10^−10^	1.78 × 10^−10^	1.21 × 10^−12^
F4	6.15 × 10^−2^	4.06 × 10^−2^	7.24 × 10^−2^	3.59 × 10^−5^	3.02 × 10^−11^	8.12 × 10^−4^	8.66 × 10^−5^	3.02 × 10^−11^
F5	1.17 × 10^−2^	8.29 × 10^−6^	7.73 × 10^−2^	3.47 × 10^−10^	3.02 × 10^−11^	1.01 × 10^−8^	2.39 × 10^−8^	7.84 × 10^−1^
F6	2.58 × 10^−1^	2.44 × 10^−9^	7.98 × 10^−2^	4.50 × 10^−11^	3.02 × 10^−11^	3.02 × 10^−11^	6.53 × 10^−7^	5.33 × 10^−10^
F7	1.17 × 10^−2^	5.97 × 10^−5^	2.77 × 10^−1^	3.02 × 10^−11^	3.02 × 10^−11^	3.02 × 10^−11^	2.75 × 10^−3^	1.11 × 10^−6^
F8	5.75 × 10^−2^	1.45 × 10^−1^	7.39 × 10^−1^	1.32 × 10^−4^	6.07 × 10^−11^	4.20 × 10^−10^	1.73 × 10^−7^	3.27 × 10^−3^
F9	1.17 × 10^−4^	1.33 × 10^−10^	2.39 × 10^−4^	3.02 × 10^−11^	3.02 × 10^−11^	3.02 × 10^−11^	6.70 × 10^−11^	3.01 × 10^−11^
F10	6.35 × 10^−2^	9.05 × 10^−2^	8.77 × 10^−1^	8.12 × 10^−4^	9.92 × 10^−11^	1.75 × 10^−5^	4.03 × 10^−3^	1.36 × 10^−7^
F11	2.92 × 10^−2^	4.64 × 10^−1^	7.60 × 10^−7^	7.22 × 10^−6^	3.02 × 10^−11^	2.20 × 10^−7^	1.56 × 10^−8^	4.57 × 10^−9^
F12	4.73 × 10^−1^	4.46 × 10^−1^	4.51 × 10^−2^	9.59 × 10^−1^	3.02 × 10^−11^	4.29 × 10^−1^	5.61 × 10^−5^	3.02 × 10^−11^
F13	8.53 × 10^−1^	4.20 × 10^−1^	1.41 × 10^−1^	7.84 × 10^−1^	3.34 × 10^−11^	8.65 × 10^−1^	2.84 × 10^−1^	3.02 × 10^−11^
F14	5.49 × 10^−1^	1.67 × 10^−1^	1.49 × 10^−4^	7.66 × 10^−5^	3.18 × 10^−1^	1.32 × 10^−4^	1.06 × 10^−3^	6.70 × 10^−11^
F15	1.76 × 10^−1^	2.92 × 10^−9^	3.52 × 10^−7^	2.78 × 10^−7^	1.78 × 10^−10^	1.21 × 10^−10^	8.20 × 10^−7^	3.02 × 10^−11^
F16	8.77 × 10^−2^	1.11 × 10^−6^	2.62 × 10^−3^	1.29 × 10^−9^	3.02 × 10^−11^	8.89 × 10^−10^	5.26 × 10^−4^	1.07 × 10^−9^
F17	2.81 × 10^−2^	6.00 × 10^−1^	3.64 × 10^−2^	1.73 × 10^−7^	4.50 × 10^−11^	1.56 × 10^−8^	3.83 × 10^−5^	1.15 × 10^−1^
F18	3.26 × 10^−1^	1.12 × 10^−2^	1.02 × 10^−1^	3.27 × 10^−2^	3.02 × 10^−11^	5.01 × 10^−2^	1.30 × 10^−1^	3.02 × 10^−11^
F19	1.22 × 10^−1^	1.04 × 10^−4^	5.56 × 10^−4^	1.75 × 10^−5^	3.02 × 10^−11^	3.18 × 10^−4^	2.78 × 10^−7^	3.02 × 10^−11^
F20	2.23 × 10^−1^	4.46 × 10^−4^	7.73 × 10^−1^	1.09 × 10^−5^	5.57 × 10^−10^	1.09 × 10^−5^	2.16 × 10^−3^	3.02 × 10^−11^
F21	3.59 × 10^−5^	1.85 × 10^−8^	1.31 × 10^−8^	3.02 × 10^−11^	4.98 × 10^−11^	9.06 × 10^−8^	6.70 × 10^−11^	3.02 × 10^−11^
F22	9.05 × 10^−2^	7.73 × 10^−1^	1.54 × 10^−1^	1.17 × 10^−9^	3.02 × 10^−11^	1.07 × 10^−9^	5.56 × 10^−4^	3.78 × 10^−10^
F23	7.17 × 10^−1^	3.65 × 10^−8^	7.06 × 10^−1^	9.92 × 10^−11^	3.02 × 10^−11^	4.50 × 10^−11^	1.27 × 10^−2^	1.86 × 10^−1^
F24	4.18 × 10^−9^	6.70 × 10^−11^	1.07 × 10^−9^	5.49 × 10^−11^	7.39 × 10^−11^	3.69 × 10^−11^	1.96 × 10^−10^	4.83 × 10^−8^
F25	8.24 × 10^−2^	6.63 × 10^−1^	3.87 × 10^−1^	1.60 × 10^−7^	3.02 × 10^−11^	1.34 × 10^−5^	1.17 × 10^−5^	1.95 × 10^−3^
F26	1.38 × 10^−2^	1.49 × 10^−4^	1.33 × 10^−1^	5.00 × 10^−9^	8.15 × 10^−11^	3.82 × 10^−10^	9.79 × 10^−5^	2.38 × 10^−8^
F27	1.37 × 10^−1^	3.69 × 10^−11^	2.60 × 10^−5^	3.02 × 10^−11^	3.02 × 10^−11^	3.02 × 10^−11^	1.61 × 10^−10^	1.62 × 10^−5^
F28	2.51 × 10^−2^	1.17 × 10^−4^	1.01 × 10^−8^	2.57 × 10^−7^	3.02 × 10^−11^	8.20 × 10^−7^	2.96 × 10^−5^	5.30 × 10^−1^
F29	6.10 × 10^−1^	1.58 × 10^−4^	7.24 × 10^−2^	3.96 × 10^−8^	5.49 × 10^−11^	7.39 × 10^−11^	1.56 × 10^−8^	7.09 × 10^−8^
F30	5.20 × 10^−1^	3.03 × 10^−2^	2.23 × 10^−1^	4.36 × 10^−2^	1.78 × 10^−10^	3.18 × 10^−4^	2.92 × 10^−2^	3.02 × 10^−11^

**Table 6:** Test results on CEC2017 for high-dimensional functions with dimension 100 (unimodal and simple multi-modal functions).

Function	Criteria	CGBPO	PO	HHO	AO	FOX	BWO	GOOSE	WOA	CMA-ES
F1	min	7.57 × 10^10^	1.44 × 10^11^	9.65 × 10^10^	1.08 × 10^11^	7.64 × 10^10^	2.73 × 10^11^	8.03 × 10^10^	1.10 × 10^11^	2.94 × 10^11^
	std	1.52 × 10^10^	1.18 × 10^10^	1.10 × 10^10^	1.22 × 10^10^	4.05 × 10^9^	4.74 × 10^9^	9.07 × 10^10^	1.48 × 10^10^	1.50 × 10^9^
	avg	8.78 × 10^10^	1.67 × 10^11^	1.21 × 10^11^	1.30 × 10^11^	9.27 × 10^10^	2.85 × 10^11^	1.43 × 10^11^	1.42 × 10^11^	2.97 × 10^11^
F3	min	2.92 × 10^5^	3.03 × 10^5^	3.38 × 10^5^	3.46 × 10^5^	6.43 × 10^5^	3.71 × 10^5^	6.82 × 10^5^	5.83 × 10^5^	1.89 × 10^13^
	std	2.48 × 10^4^	1.79 × 10^4^	9.13 × 10^4^	5.80 × 10^3^	1.29 × 10^5^	6.97 × 10^8^	1.31 × 10^5^	1.01 × 10^5^	5.74 × 10^13^
	avg	3.31 × 10^5^	3.43 × 10^5^	3.75 × 10^5^	3.63 × 10^5^	8.60 × 10^5^	1.92 × 10^8^	8.70 × 10^5^	9.19 × 10^5^	1.05 × 10^14^
F4	min	1.29 × 10^4^	1.76 × 10^4^	1.37 × 10^4^	2.53 × 10^4^	1.49 × 10^4^	1.16 × 10^5^	1.53 × 10^4^	2.31 × 10^4^	1.55 × 10^5^
	std	3.18 × 10^3^	5.68 × 10^3^	3.43 × 10^3^	4.41 × 10^3^	1.26 × 10^3^	1.07 × 10^4^	2.82 × 10^4^	5.28 × 10^3^	1.75 × 10^3^
	avg	1.84 × 10^4^	2.94 × 10^4^	2.08 × 10^4^	3.25 × 10^4^	1.71 × 10^4^	1.34 × 10^5^	4.23 × 10^4^	3.23 × 10^4^	1.58 × 10^5^
F5	min	1.33 × 10^3^	1.79 × 10^3^	1.63 × 10^3^	1.68 × 10^3^	1.33 × 10^3^	2.10 × 10^3^	1.71 × 10^3^	1.85 × 10^3^	2.28 × 10^3^
	std	6.86 × 10^1^	6.20 × 10^1^	5.65 × 10^1^	6.69 × 10^1^	2.29 × 10^1^	3.29 × 10^1^	4.18 × 10^2^	7.06 × 10^1^	3.06 × 10^1^
	avg	1.84 × 10^3^	1.90 × 10^3^	1.74 × 10^3^	1.81 × 10^3^	1.38 × 10^3^	2.16 × 10^3^	1.80 × 10^3^	1.99 × 10^3^	2.34 × 10^3^
F6	min	6.84 × 10^2^	6.81 × 10^2^	6.86 × 10^2^	6.87 × 10^2^	6.66 × 10^2^	7.10 × 10^2^	6.65 × 10^2^	6.95 × 10^2^	7.26 × 10^2^
	std	5.67 × 10^0^	6.35 × 10^0^	3.94 × 10^0^	4.96 × 10^0^	1.79 × 10^0^	1.96 × 10^0^	1.31 × 10^1^	1.03 × 10^1^	3.70 × 10^0^
	avg	6.70 × 10^2^	6.99 × 10^2^	6.93 × 10^2^	6.95 × 10^2^	6.92 × 10^2^	7.15 × 10^2^	6.80 × 10^2^	7.11 × 10^2^	7.35 × 10^2^
F7	min	3.49 × 10^3^	3.52 × 10^3^	3.55 × 10^3^	3.25 × 10^3^	3.20 × 10^3^	3.99 × 10^3^	3.31 × 10^3^	3.55 × 10^3^	4.17 × 10^3^
	std	1.04 × 10^2^	1.02 × 10^2^	1.03 × 10^2^	1.54 × 10^2^	6.37 × 10^1^	4.84 × 10^1^	4.11 × 10^3^	1.36 × 10^2^	5.36 × 10^1^
	avg	3.34 × 10^3^	3.74 × 10^3^	3.81 × 10^3^	3.60 × 10^3^	3.67 × 10^3^	4.12 × 10^3^	7.00 × 10^3^	3.86 × 10^3^	4.27 × 10^3^
F8	min	2.15 × 10^3^	2.13 × 10^3^	1.96 × 10^3^	2.04 × 10^3^	1.83 × 10^3^	2.60 × 10^3^	1.85 × 10^3^	2.22 × 10^3^	2.75 × 10^3^
	std	7.75 × 10^1^	7.84 × 10^1^	7.18 × 10^1^	6.35 × 10^1^	2.43 × 10^1^	2.79 × 10^1^	3.79 × 10^2^	1.40 × 10^2^	2.88 × 10^1^
	avg	1.89 × 10^3^	2.34 × 10^3^	2.20 × 10^3^	2.22 × 10^3^	2.29 × 10^3^	2.66 × 10^3^	2.29 × 10^3^	2.48 × 10^3^	2.81 × 10^3^
F9	min	5.19 × 10^4^	4.99 × 10^4^	5.59 × 10^4^	5.48 × 10^4^	3.15 × 10^4^	7.28 × 10^4^	2.98 × 10^4^	5.99 × 10^4^	9.00 × 10^4^
	std	1.48 × 10^3^	7.00 × 10^3^	6.03 × 10^3^	6.68 × 10^3^	5.62 × 10^3^	3.10 × 10^3^	1.73 × 10^4^	1.84 × 10^4^	8.79 × 10^3^
	avg	6.28 × 10^4^	6.38 × 10^4^	7.32 × 10^4^	6.85 × 10^4^	3.46 × 10^4^	8.06 × 10^4^	5.81 × 10^4^	8.44 × 10^4^	1.07 × 10^5^
F10	min	2.53 × 10^4^	2.70 × 10^4^	2.20 × 10^4^	2.36 × 10^4^	1.54 × 10^4^	3.11 × 10^4^	1.54 × 10^4^	2.82 × 10^4^	3.47 × 10^4^
	std	1.37 × 10^3^	1.37 × 10^3^	1.53 × 10^3^	1.74 × 10^3^	1.07 × 10^3^	5.68 × 10^2^	1.29 × 10^3^	1.06 × 10^3^	6.76 × 10^2^
	avg	2.82 × 10^4^	2.91 × 10^4^	2.52 × 10^4^	2.67 × 10^4^	1.74 × 10^4^	3.29 × 10^4^	1.77 × 10^4^	3.03 × 10^4^	3.63 × 10^4^
F11	min	8.15 × 10^4^	9.87 × 10^4^	8.40 × 10^4^	2.44 × 10^5^	1.38 × 10^5^	2.06 × 10^5^	1.65 × 10^5^	1.22 × 10^5^	2.17 × 10^13^
	std	2.03 × 10^4^	2.09 × 10^4^	3.65 × 10^4^	1.17 × 10^5^	6.69 × 10^4^	5.77 × 10^4^	8.43 × 10^4^	1.36 × 10^5^	4.53 × 10^11^
	avg	1.23 × 10^5^	1.36 × 10^5^	1.95 × 10^5^	4.41 × 10^5^	2.74 × 10^5^	3.53 × 10^5^	3.02 × 10^5^	3.24 × 10^5^	2.34 × 10^13^

**Table 7:** Test results on CEC2017 for high-dimensional functions with dimension 100 (hybrid functions).

Function	Criteria	CGBPO	PO	HHO	AO	FOX	BWO	GOOSE	WOA	CMA-ES
F12	min	1.56 × 10^10^	4.07 × 10^10^	1.68 × 10^10^	4.12 × 10^10^	3.00 × 10^10^	2.02 × 10^11^	3.23 × 10^10^	3.54 × 10^10^	2.55 × 10^11^
	std	8.64 × 10^9^	1.34 × 10^10^	1.22 × 10^10^	1.16 × 10^10^	3.06 × 10^9^	1.43 × 10^10^	2.81 × 10^10^	1.22 × 10^10^	1.91 × 10^9^
	avg	3.38 × 10^10^	6.22 × 10^10^	3.75 × 10^10^	6.39 × 10^10^	3.56 × 10^10^	2.31 × 10^11^	6.46 × 10^10^	5.22 × 10^10^	2.59 × 10^11^
F13	min	1.11 × 10^9^	6.40 × 10^9^	8.62 × 10^8^	4.61 × 10^9^	1.50 × 10^9^	4.69 × 10^10^	1.52 × 10^9^	3.00 × 10^9^	6.42 × 10^10^
	std	1.53 × 10^9^	3.58 × 10^9^	1.68 × 10^9^	2.08 × 10^9^	7.43 × 10^8^	4.23 × 10^9^	4.94 × 10^9^	2.07 × 10^9^	5.14 × 10^8^
	avg	4.09 × 10^9^	1.21 × 10^10^	3.57 × 10^9^	8.83 × 10^9^	2.70 × 10^9^	5.56 × 10^10^	8.30 × 10^9^	6.26 × 10^9^	6.53 × 10^10^
F14	min	2.67 × 10^6^	1.07 × 10^7^	7.47 × 10^6^	9.89 × 10^6^	3.12 × 10^6^	8.63 × 10^7^	1.61 × 10^6^	9.00 × 10^6^	1.32 × 10^9^
	std	5.79 × 10^6^	7.05 × 10^6^	6.41 × 10^6^	1.42 × 10^7^	9.17 × 10^6^	1.28 × 10^8^	1.17 × 10^7^	1.37 × 10^7^	6.36 × 10^7^
	avg	1.06 × 10^7^	1.99 × 10^7^	1.45 × 10^7^	2.79 × 10^7^	1.56 × 10^7^	2.94 × 10^8^	1.46 × 10^7^	2.91 × 10^7^	1.43 × 10^9^
F15	min	1.58 × 10^4^	2.06 × 10^8^	6.73 × 10^7^	9.10 × 10^8^	2.58 × 10^7^	2.14 × 10^10^	3.08 × 10^8^	4.99 × 10^8^	4.04 × 10^10^
	std	5.34 × 10^8^	2.32 × 10^9^	5.46 × 10^8^	1.04 × 10^9^	2.02 × 10^7^	4.61 × 10^9^	1.04 × 10^9^	5.88 × 10^8^	4.02 × 10^8^
	avg	7.45 × 10^7^	3.23 × 10^9^	5.87 × 10^8^	2.64 × 10^9^	1.10 × 10^8^	3.25 × 10^10^	9.11 × 10^8^	1.29 × 10^9^	4.11 × 10^10^
F16	min	1.58 × 10^7^	2.06 × 10^8^	6.73 × 10^7^	9.10 × 10^8^	2.58 × 10^7^	2.14 × 10^10^	3.08 × 10^8^	4.99 × 10^8^	4.04 × 10^10^
	std	5.34 × 10^8^	2.32 × 10^9^	5.46 × 10^8^	1.04 × 10^9^	2.02 × 10^7^	4.61 × 10^9^	1.04 × 10^9^	5.88 × 10^8^	4.02 × 10^8^
	avg	7.45 × 10^7^	3.23 × 10^9^	5.87 × 10^8^	2.64 × 10^9^	1.10 × 10^8^	3.25 × 10^10^	9.11 × 10^8^	1.29 × 10^9^	4.11 × 10^10^
F17	min	5.30 × 10^3^	7.47 × 10^3^	6.06 × 10^3^	1.23 × 10^4^	7.19 × 10^3^	3.67 × 10^6^	5.87 × 10^3^	8.83 × 10^3^	1.52 × 10^8^
	std	1.84 × 10^3^	5.53 × 10^4^	7.58 × 10^4^	6.78 × 10^4^	8.55 × 10^3^	2.26 × 10^7^	6.17 × 10^3^	6.67 × 10^4^	9.97 × 10^6^
	avg	1.10 × 10^4^	5.60 × 10^4^	1.41 × 10^4^	7.93 × 10^4^	1.01 × 10^4^	3.55 × 10^7^	1.93 × 10^4^	4.92 × 10^4^	1.72 × 10^8^
F18	min	1.36 × 10^6^	6.07 × 10^6^	2.24 × 10^6^	4.92 × 10^7^	1.53 × 10^6^	1.34 × 10^8^	1.98 × 10^6^	5.07 × 10^6^	1.33 × 10^9^
	std	6.74 × 10^6^	1.49 × 10^7^	7.52 × 10^6^	1.47 × 10^7^	9.57 × 10^6^	2.71 × 10^8^	9.29 × 10^6^	1.22 × 10^7^	6.09 × 10^7^
	avg	1.64 × 10^6^	2.80 × 10^7^	1.21 × 10^7^	3.08 × 10^7^	6.52 × 10^7^	7.23 × 10^8^	9.59 × 10^6^	2.26 × 10^7^	1.44 × 10^9^
F19	min	2.57 × 10^8^	1.21 × 10^9^	1.11 × 10^8^	7.17 × 10^8^	9.75 × 10^5^	2.03 × 10^10^	1.11 × 10^6^	4.96 × 10^8^	4.06 × 10^10^
	std	3.96 × 10^7^	2.17 × 10^9^	3.38 × 10^8^	9.36 × 10^8^	5.89 × 10^8^	3.51 × 10^9^	1.20 × 10^9^	5.74 × 10^8^	4.31 × 10^8^
	avg	6.88 × 10^8^	3.62 × 10^9^	4.54 × 10^8^	2.35 × 10^9^	3.22 × 10^7^	3.38 × 10^10^	8.59 × 10^8^	1.31 × 10^9^	4.15 × 10^10^
F20	min	5.33 × 10^3^	6.03 × 10^3^	5.35 × 10^3^	5.47 × 10^3^	4.79 × 10^3^	7.20 × 10^3^	5.54 × 10^3^	6.45 × 10^3^	9.91 × 10^3^
	std	5.04 × 10^2^	5.68 × 10^2^	4.34 × 10^2^	4.84 × 10^2^	5.81 × 10^2^	3.24 × 10^2^	6.06 × 10^2^	6.16 × 10^2^	1.38 × 10^2^
	avg	6.40 × 10^3^	6.95 × 10^3^	6.37 × 10^3^	6.35 × 10^3^	6.46 × 10^3^	7.99 × 10^3^	6.51 × 10^3^	7.39 × 10^3^	1.04 × 10^4^
F21	min	3.69 × 10^3^	3.86 × 10^3^	3.98 × 10^3^	3.89 × 10^3^	3.91 × 10^3^	4.97 × 10^3^	4.09 × 10^3^	3.94 × 10^3^	7.35 × 10^3^
	std	1.41 × 10^2^	1.46 × 10^2^	1.97 × 10^2^	3.10 × 10^2^	2.67 × 10^2^	1.36 × 10^2^	2.38 × 10^2^	2.49 × 10^2^	5.55 × 10^−12^
	avg	4.05 × 10^3^	4.12 × 10^3^	4.50 × 10^3^	4.42 × 10^3^	4.50 × 10^3^	5.22 × 10^3^	4.52 × 10^3^	4.53 × 10^3^	7.35 × 10^3^

**Table 8:** Test results on CEC2017 for high-dimensional functions with dimension 100 (composition functions and extended unimodal functions).

Function	Criteria	CGBPO	PO	HHO	AO	FOX	BWO	GOOSE	WOA	CMA-ES
F22	min	2.54 × 10^4^	2.84 × 10^4^	2.73 × 10^4^	2.85 × 10^4^	1.68 × 10^4^	3.43 × 10^4^	1.82 × 10^4^	2.89 × 10^4^	3.86 × 10^4^
	std	2.02 × 10^3^	1.33 × 10^3^	1.17 × 10^3^	1.11 × 10^3^	1.63 × 10^3^	4.53 × 10^2^	1.79 × 10^3^	1.36 × 10^3^	7.37 × 10^2^
	avg	3.06 × 10^4^	3.18 × 10^4^	2.90 × 10^4^	2.99 × 10^4^	2.05 × 10^4^	3.51 × 10^4^	2.08 × 10^4^	3.26 × 10^4^	4.04 × 10^4^
F23	min	4.53 × 10^3^	4.61 × 10^3^	5.38 × 10^3^	4.53 × 10^3^	5.13 × 10^3^	7.01 × 10^3^	5.23 × 10^3^	4.94 × 10^3^	8.42 × 10^3^
	std	2.19 × 10^2^	2.18 × 10^2^	5.22 × 10^2^	2.79 × 10^2^	4.30 × 10^2^	4.49 × 10^2^	3.53 × 10^2^	2.43 × 10^2^	5.55 × 10^−12^
	avg	4.92 × 10^3^	4.93 × 10^3^	6.05 × 10^3^	5.15 × 10^3^	6.07 × 10^3^	7.95 × 10^3^	5.95 × 10^3^	5.44 × 10^3^	8.42 × 10^3^
F24	min	5.76 × 10^3^	5.82 × 10^3^	7.31 × 10^3^	6.20 × 10^3^	8.17 × 10^3^	1.18 × 10^4^	8.09 × 10^3^	6.46 × 10^3^	1.42 × 10^4^
	std	3.32 × 10^2^	4.14 × 10^2^	6.95 × 10^2^	4.62 × 10^2^	4.39 × 10^2^	1.19 × 10^3^	6.12 × 10^2^	3.73 × 10^2^	7.40 × 10^−12^
	avg	6.31 × 10^3^	6.60 × 10^3^	8.76 × 10^3^	7.15 × 10^3^	8.94 × 10^3^	1.43 × 10^4^	9.03 × 10^3^	6.95 × 10^3^	1.42 × 10^4^
F25	min	8.61 × 10^3^	1.15 × 10^4^	7.97 × 10^3^	9.97 × 10^3^	7.28 × 10^3^	2.84 × 10^4^	7.70 × 10^3^	1.13 × 10^4^	3.47 × 10^4^
	std	1.35 × 10^3^	1.77 × 10^3^	1.20 × 10^3^	1.30 × 10^3^	3.20 × 10^2^	1.44 × 10^3^	1.38 × 10^4^	1.35 × 10^3^	5.51 × 10^2^
	avg	8.13 × 10^3^	1.55 × 10^4^	9.92 × 10^3^	1.28 × 10^4^	1.04 × 10^4^	3.27 × 10^4^	2.33 × 10^4^	1.41 × 10^4^	3.55 × 10^4^
F26	min	2.65 × 10^4^	3.06 × 10^4^	2.90 × 10^4^	3.24 × 10^4^	2.82 × 10^4^	5.31 × 10^4^	2.88 × 10^4^	3.50 × 10^4^	6.52 × 10^4^
	std	3.00 × 10^3^	2.68 × 10^3^	2.29 × 10^3^	1.86 × 10^3^	8.80 × 10^2^	1.83 × 10^3^	1.03 × 10^4^	2.93 × 10^3^	3.91 × 10^2^
	avg	3.00 × 10^4^	3.89 × 10^4^	3.41 × 10^4^	3.67 × 10^4^	3.60 × 10^4^	5.87 × 10^4^	3.90 × 10^4^	3.99 × 10^4^	6.59 × 10^4^
F27	min	4.65 × 10^3^	4.85 × 10^3^	5.38 × 10^3^	6.97 × 10^3^	6.52 × 10^3^	1.36 × 10^4^	6.42 × 10^3^	5.26 × 10^3^	2.48 × 10^4^
	std	7.11 × 10^2^	7.70 × 10^2^	1.37 × 10^3^	6.68 × 10^2^	1.78 × 10^3^	1.34 × 10^3^	1.76 × 10^3^	1.17 × 10^3^	2.51 × 10^2^
	avg	5.74 × 10^3^	6.00 × 10^3^	7.53 × 10^3^	8.38 × 10^3^	9.65 × 10^3^	1.63 × 10^4^	9.58 × 10^3^	6.94 × 10^3^	2.54 × 10^4^
F28	min	1.16 × 10^4^	1.60 × 10^4^	1.11 × 10^4^	1.47 × 10^4^	1.25 × 10^4^	3.11 × 10^4^	1.46 × 10^4^	1.51 × 10^4^	4.27 × 10^4^
	std	1.65 × 10^3^	1.57 × 10^3^	9.16 × 10^2^	1.72 × 10^3^	5.94 × 10^2^	1.07 × 10^3^	7.19 × 10^3^	1.14 × 10^3^	3.62 × 10^2^
	avg	1.60 × 10^4^	1.89 × 10^4^	1.31 × 10^4^	1.79 × 10^4^	1.35 × 10^4^	3.29 × 10^4^	2.64 × 10^4^	1.79 × 10^4^	4.33 × 10^4^
F29	min	1.16 × 10^4^	1.49 × 10^4^	1.35 × 10^4^	1.65 × 10^4^	1.20 × 10^4^	4.74 × 10^5^	1.20 × 10^4^	1.70 × 10^4^	7.34 × 10^6^
	std	1.20 × 10^3^	1.31 × 10^4^	1.94 × 10^3^	9.56 × 10^3^	4.37 × 10^3^	8.06 × 10^5^	1.43 × 10^4^	7.27 × 10^3^	5.06 × 10^5^
	avg	1.31 × 10^4^	3.10 × 10^4^	1.86 × 10^4^	2.89 × 10^4^	1.59 × 10^4^	1.90 × 10^6^	2.24 × 10^4^	2.58 × 10^4^	8.38 × 10^6^
F30	min	1.22 × 10^9^	3.20 × 10^9^	1.28 × 10^9^	4.62 × 10^9^	1.43 × 10^9^	4.06 × 10^0^	1.80 × 10^9^	2.42 × 10^9^	5.95 × 10^0^
	std	1.33 × 10^9^	3.28 × 10^9^	1.61 × 10^9^	2.23 × 10^9^	1.02 × 10^9^	4.80 × 10^9^	3.93 × 10^9^	2.10 × 10^9^	5.85 × 10^8^
	avg	3.13 × 10^9^	9.77 × 10^9^	3.38 × 10^9^	8.44 × 10^9^	4.79 × 10^9^	5.06 × 10^0^	6.97 × 10^9^	5.24 × 10^9^	6.07 × 10^0^

**Table 9:** Test results on CEC2022.

Function	Criteria	CGBPO	PO	HHO	AO	FOX	BWO	GOOSE	WOA	CMA-ES
F1	min	325.52	355.75	1069.21	2349.95	385.19	11,193.72	1783.09	11,849.54	300.00
	std	280.32	1049.90	1304.51	2719.52	8462.17	16,636.39	10,576.64	13,601.11	0.00
	avg	620.23	1058.95	3038.21	6366.09	14,786.70	32,693.97	15,310.28	28,148.44	300.00
F2	min	406.11	404.48	400.25	401.69	400.02	885.63	400.03	411.90	400.00
	std	26.26	32.19	72.18	56.08	165.15	1010.27	106.39	41.97	2.37
	avg	441.81	452.71	474.15	467.09	558.96	2377.61	513.78	474.28	407.80
F3	min	607.97	609.34	619.88	609.13	635.91	632.74	628.56	612.25	608.81
	std	7.18	11.38	12.05	8.37	9.49	10.97	14.96	16.70	9.87
	avg	624.54	626.79	641.84	623.54	654.60	660.93	656.53	645.33	655.49
F4	min	812.96	816.17	811.24	810.26	821.89	843.08	816.91	817.79	828.85
	std	7.15	6.45	6.41	7.01	4.77	6.28	17.95	13.79	0.81
	avg	823.54	831.40	825.92	825.61	832.77	855.14	852.68	843.79	832.44
F5	min	916.66	959.82	1025.37	931.88	1435.96	1377.33	1381.03	1098.63	1425.20
	std	123.80	196.77	197.33	133.64	85.64	185.84	557.80	561.01	13.44
	avg	1019.74	1208.81	1467.60	1089.63	1484.54	1745.06	2032.22	1674.67	1459.28
F6	min	1956.99	1883.56	2035.15	3559.25	1889.86	17,422,511.16	1997.97	2280.29	1800.36
	std	2213.76	2351.93	6096.25	85,201.85	2023.99	672,783,088.06	2368.52	24,520.47	6.06
	avg	4354.42	4572.60	9321.28	67,141.02	4379.85	1,015,177,368.97	4464.96	16,555.45	1807.81
F7	min	2003.98	2027.32	2048.69	2031.87	2077.88	2029.84	2059.45	2040.18	2094.92
	std	14.19	17.17	30.29	17.47	56.33	18.49	87.86	28.80	92.59
	avg	2055.09	2066.57	2092.73	2055.46	2151.88	2135.21	2185.77	2088.42	2288.13
F8	min	2216.14	2223.61	2209.77	2227.42	2224.13	2237.67	2224.85	2221.67	2201.71
	std	5.36	5.89	12.90	4.41	137.79	26.95	140.15	25.02	26.97
	avg	2229.93	2232.01	2234.72	2232.85	2358.08	2276.56	2438.53	2243.10	2226.92
F9	min	2529.86	2546.97	2542.16	2568.95	2553.72	2701.75	2569.87	2539.11	2529.28
	std	38.37	44.83	45.63	37.40	63.61	41.77	52.96	51.03	0.00
	avg	2581.28	2609.89	2641.10	2631.16	2676.27	2778.04	2657.18	2631.48	2529.28
F10	min	2500.44	2500.72	2500.62	2500.96	2500.99	2582.11	2500.68	2500.65	2604.91
	std	0.86	57.94	210.94	57.11	651.13	283.78	481.74	267.94	2.42
	avg	2501.38	2536.22	2637.54	2587.76	3209.99	2881.40	2955.98	2646.60	2608.25
F11	min	2674.68	2706.49	2612.50	2661.30	2715.42	3034.73	2725.87	2739.26	2600.00
	std	25.55	79.65	198.51	109.34	387.75	462.46	30,599.07	167.34	134.93
	avg	2747.09	2801.13	2889.72	2784.05	3333.11	3673.11	31,826.27	3091.88	2820.00
F12	min	2863.50	2863.60	2864.61	2866.04	2901.59	2895.73	2881.81	2865.46	2862.70
	std	5.25	12.17	64.60	10.39	119.98	73.62	100.61	57.31	1.06
	avg	2867.53	2871.59	2926.29	2873.24	3040.99	3028.49	3011.19	2914.78	2865.22

**Table 10:** *p*-values from Wilcoxon’s rank-sum test on CEC2022.

Function	PO	HHO	AO	FOX	BWO	GOOSE	WOA	CMA-ES
F1	8.24 × 10^−2^	5.49 × 10^−11^	3.02 × 10^−11^	4.62 × 10^−10^	3.02 × 10^−11^	3.02 × 10^−11^	3.02 × 10^−11^	1.21 × 10^−12^
F2	1.91 × 10^−1^	2.32 × 10^−2^	2.24 × 10^−2^	7.20 × 10^−5^	3.02 × 10^−11^	9.52 × 10^−4^	8.56 × 10^−4^	2.90 × 10^−9^
F3	7.28 × 10^−1^	3.26 × 10^−7^	6.31 × 10^−1^	6.07 × 10^−11^	5.49 × 10^−11^	3.82 × 10^−10^	6.05 × 10^−7^	4.81 × 10^−10^
F4	4.35 × 10^−5^	1.30 × 10^−1^	1.76 × 10^−1^	1.19 × 10^−6^	3.02 × 10^−11^	2.44 × 10^−9^	2.20 × 10^−7^	1.59 × 10^−7^
F5	5.46 × 10^−6^	4.20 × 10^−10^	6.55 × 10^−4^	4.62 × 10^−10^	4.50 × 10^−11^	8.15 × 10^−11^	4.20 × 10^−10^	5.55 × 10^−10^
F6	9.35 × 10^−1^	4.22 × 10^−4^	1.29 × 10^−9^	8.88 × 10^−1^	3.02 × 10^−11^	8.88 × 10^−1^	3.56 × 10^−4^	3.02 × 10^−11^
F7	7.62 × 10^−3^	2.78 × 10^−7^	7.28 × 10^−1^	3.34 × 10^−11^	3.02 × 10^−11^	2.37 × 10^−10^	4.12 × 10^−6^	5.57 × 10^−10^
F8	2.64 × 10^−1^	1.96 × 10^−1^	1.84 × 10^−2^	8.15 × 10^−5^	4.98 × 10^−11^	6.72 × 10^−10^	1.58 × 10^−4^	1.61 × 10^−6^
F9	1.17 × 10^−2^	5.46 × 10^−6^	3.16 × 10^−5^	1.07 × 10^−7^	3.02 × 10^−11^	8.20 × 10^−7^	1.41 × 10^−4^	3.02 × 10^−11^
F10	3.85 × 10^−3^	1.17 × 10^−5^	1.55 × 10^−9^	6.72 × 10^−10^	3.02 × 10^−11^	9.06 × 10^−8^	2.24 × 10^−2^	3.02 × 10^−11^
F11	2.00 × 10^−5^	2.75 × 10^−3^	4.20 × 10^−1^	4.62 × 10^−10^	3.02 × 10^−11^	5.07 × 10^−10^	5.46 × 10^−9^	1.95 × 10^−3^
F12	9.93 × 10^−2^	1.07 × 10^−9^	2.49 × 10^−6^	3.02 × 10^−11^	3.02 × 10^−11^	3.34 × 10^−11^	1.31 × 10^−8^	5.31 × 10^−3^

**Table 11:** Test results regarding the design optimization problem for industrial refrigeration systems.

Function	CGBPO	PO	HHO	AO	FOX	BWO	GOOSE	WOA	CMA_ES
min	5.32 × 10^−1^	1.11 × 10^0^	2.32 × 10^2^	5.01 × 10^3^	4.93 × 10^0^	3.04 × 10^16^	8.62 × 10^0^	2.23 × 10^1^	1.09 × 10^0^
std	2.96 × 10^14^	3.01 × 10^14^	1.95 × 10^15^	6.52 × 10^15^	1.80 × 10^15^	1.38 × 10^16^	2.64 × 10^15^	1.77 × 10^16^	4.83 × 10^14^
avg	9.36 × 10^13^	9.52 × 10^13^	1.14 × 10^15^	3.74 × 10^15^	1.51 × 10^15^	4.93 × 10^16^	1.96 × 10^15^	1.00 × 10^16^	5.62 × 10^14^

**Table 12:** Test results for Himmel Blau’s function optimization problem.

Function	CGBPO	WOA	PO	HHO	AO	FOX	BWO	GOOSE	CMA-ES
min	−30,664.6	−30,665.4	−30,651.6	−30,556	−30,665.3	−30,238.9	−30,664.4	−30,045.7	−29,782.9
std	113.3692	201.8359	263.5462	237.3315	249.8378	154.6196	225.8986	451.5172	196.1186
avg	−30,509.6	−30,505.3	−30,442.6	−30,299.2	−30,394.7	−30,068	−30,471.4	−29,477.6	−29,433

**Table 13:** Related-parameter settings.

Parameter	Value
Emitting the power of LED, $P_{t}$	2.2 W
Photo-electric conversion efficiency of PD, $R_{p}$	0.5 A/W
Equivalent noise bandwidth, B	100 Mb/s
Background photocurrent, $I_{bg}$	5100 μA
Noise bandwidth factor, $I_{2}$	0.562
Noise bandwidth factor, $I_{3}$	0.0868
Absolute temperature, $T_{k}$	295 K
Open-loop voltage gain, G	10
Transconductance of Field-Effect Transistor, $g_{m}$	30 mS
Channel noise coefficient of Field-Effect Transistor, Γ	1.5
Unit-area capacitance of PD, η	112 pF/cm^2^
Population size, $N_{p}$	100
Max iteration number, $G_{\max}$	60

## Data Availability

All the data presented in this study are available within the main text.

## Abbreviations

The following abbreviations are used in this manuscript:

DOAJ	Directory of open access journals
TLA	Three-letter acronym
LD	Linear dichroism
